# Algal Toxin Azaspiracid-1 Induces Early Neuronal Differentiation and Alters Peripherin Isoform Stoichiometry

**DOI:** 10.3390/md13127072

**Published:** 2015-12-14

**Authors:** Linda V. Hjørnevik, Ann K. Frøyset, Toril A. Grønset, Krisna Rungruangsak-Torrissen, Kari E. Fladmark

**Affiliations:** 1 Department of Molecular Biology, University of Bergen, Thormøhlensgate 55, N-5008 Bergen, Norway; Linda.Hjornevik@uib.no (L.V.H.); Ann.Froyset@uib.no (A.K.F.); Toril-Anne.Gronset@uit.no (T.A.G.); 2 Institute of Marine Research, Matre Research Station, N-5984 Matredal, Norway; Krisnart@imr.no

**Keywords:** azaspiracid, algal toxin, neurotoxin, peripherin, intermediate filament, isoform, PC12 cells

## Abstract

Azaspiracid-1 is an algal toxin that accumulates in edible mussels, and ingestion may result in human illness as manifested by vomiting and diarrhoea. When injected into mice, it causes neurotoxicological symptoms and death. Although it is well known that azaspiracid-1 is toxic to most cells and cell lines, little is known about its biological target(s). A rat PC12 cell line, commonly used as a model for the peripheral nervous system, was used to study the neurotoxicological effects of azaspiracid-1. Azaspiracid-1 induced differentiation-related morphological changes followed by a latter cell death. The differentiated phenotype showed peripherin-labelled neurite-like processes simultaneously as a specific isoform of peripherin was down-regulated. The precise mechanism behind this down-regulation remains uncertain. However, this study provides new insights into the neurological effects of azaspiracid-1 and into the biological significance of specific isoforms of peripherin.

## 1. Introduction

The algal toxin azaspiracid-1 (AZA-1) was discovered in 1995 when it caused an outbreak of diarrhetic shellfish poisoning (DSP)-like disease in humans. The symptoms included nausea, vomiting, diarrhoea and stomach cramps [1]. Oral administration of AZA-1 to mice induced histopathological changes of the intestine [2] and revealed a tumour promoting activity by the toxin [3]. Unlike DSP-causing toxins, intraperitoneal injections of AZA-1 extracts in mice produced neurological symptoms such as respiratory difficulties, spasms, paralysis and death [1,4]. Additionally, AZA-1 did not, unlike DSP-causing toxins, inhibit protein phosphatases 1 and 2A [5]. This led to the recognition that AZA-1 and its analogues produce a distinct disease named azaspiracid shellfish poisoning [6].

AZA-1 is cytotoxic in the nanomolar range to a range of different mammalian cell types [5,7,8], including neuroblastoma cells [7,8,9] and primary neurons [10,11,12]. The toxin is able to activate both necrotic and apoptotic cell death pathways [8,11]. The c-Jun-N-terminal protein kinase (JNK) has been shown to be implicated in AZA-1-induced neuronal cell death [13]. Two global studies, in which changes in the transcriptome [14] and proteome [9] after AZA-1 treatment were studied, revealed that AZA-1 induces changes in metabolic processes. However, the biological target of AZA-1 and its mode of action still remain elusive.

AZA-1 affects neuronal signalling in different cell types. The toxin is able to inhibit neurological signalling in spinal cord neuronal networks, in young cerebellar granule cell cultures and in primary neocortical neurons, and at high concentrations it acts as a hERG (human ether-à-go-go related gene) channel blocker [11,12,15,16]. In a recent study, glutarylcarnitine, the main metabolite of glutaric acid, was identified in azaspiracid-contaminated shellfish extracts [17]. Following this, Chevallier *et al.* [17] showed that in the presence of glutaric acid, nanomolar concentration of AZA-1 could inhibit the activity of sodium channels *in vitro*.

In mice orally exposed to AZA-1 the largest concentration of toxin was found in the gastrointestinal tract [18]. Since AZA-1 has been shown to have a toxic effect on neuronal cells and their derivatives [7,8,9,10,11,12], the enteric nervous system of the gastrointestinal tract might be a target for the toxin. To further elucidate the neurotoxic effect of AZA-1 we have therefore used PC12 cells, a cell line commonly used as a model for the enteric nervous system [19] and in studies focusing on intestinal neuronal-epithelial interaction [20]. AZA-1 exposure induced an early differentiation followed by cell death in PC12 cells. When elucidating markers known to be involved in PC12 differentiation, we found that AZA-1 specifically down-regulated an isoform of the neurospecific intermediate filament protein peripherin. Peripherin is mainly expressed throughout the peripheral nervous system, but it is also found in confined populations of neurons in the central nervous system, particularly in neurons involved in motor function [21,22,23,24,25].

## 2. Results

### 2.1. AZA-1 Induces Morphological Alterations in PC12 Cells

**Figure 1:**
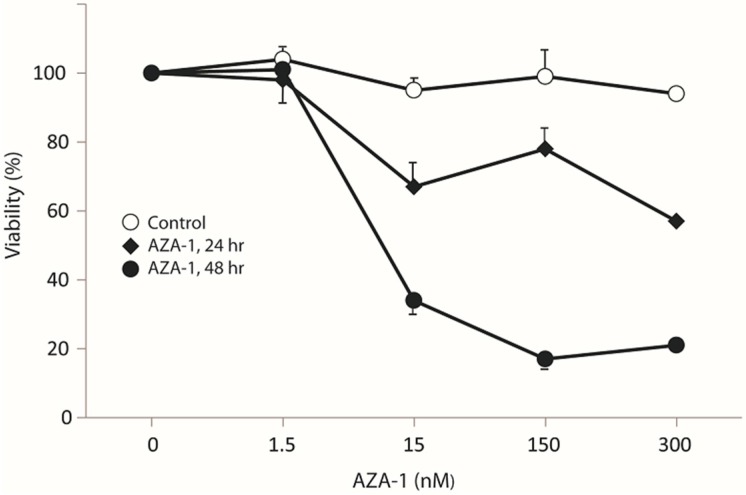
Concentration-dependent effect of AZA-1 on PC12 cell viability. PC12 cells were exposed to increasing dosages of AZA-1 or corresponding solvent control. Cell viability was determined by CCK-8 assay. Values are the mean ± SD (*n* = 2–3).

In our initial dose-response experiments, PC12 cells exposed to 15 nM showed a significant reduction in cell viability after 24 h as compared to control (Figure 1).

**Figure 2:**
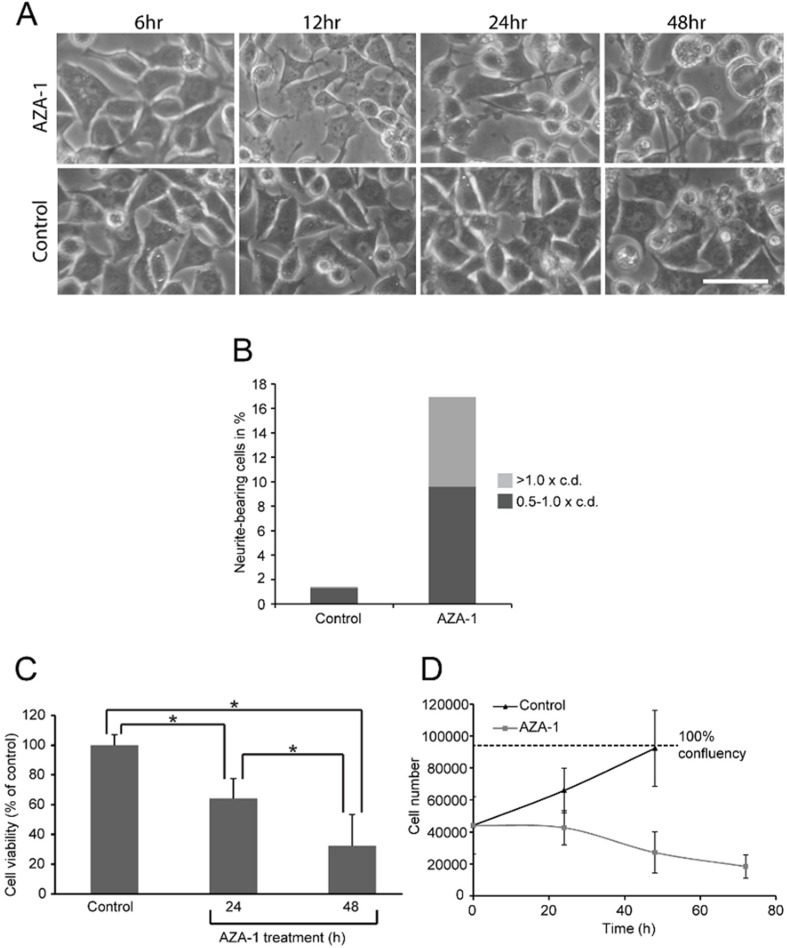
Effect of AZA-1 on PC12 morphology and cell viability. PC12 cells were treated with 15 nM AZA-1 for 6 h to 72 h, or negative control. (**A**) Cell morphology after AZA-1 exposure. AZA-treated cells appeared more differentiated with neurite-like protrusions compared to controls. Bar, 20 μm. (**B**) Quantitation of neurite-like protrusions. C.d. = cell diameter. (**C**) Cell viability as determined based on dehydrogenase activity using CCK-8 assay. Data are presented as mean ± SD (*n* = 9) of three independent experiments. Statistical analysis was performed using one-way ANOVA followed by Tukey’s *post hoc* test (* *p* < 0.01). (**D**) Reduction of cell number after AZA-1 exposure. Data are shown as mean ± SD (*n* = 3).

Further experiments were therefore performed using 15 nM AZA-1 or solvent control for 24–72 h. AZA-1-exposed cells had a more rounded morphology and neurite-like outgrowths when compared to control cells (Figure 2A,B).

Approx. 17% of the AZA-1-treated cells were scored positive for neurite-like outgrowths (Figure 2B). However, at 48 h of exposure, the viability and cell number in AZA-1-treated cells were reduced, although some cells seemed to survive AZA-1 treatment (Figure 2C,D).

### 2.2. AZA-1 Down-Regulates a Specific Peripherin Isoform

**Figure 3:**
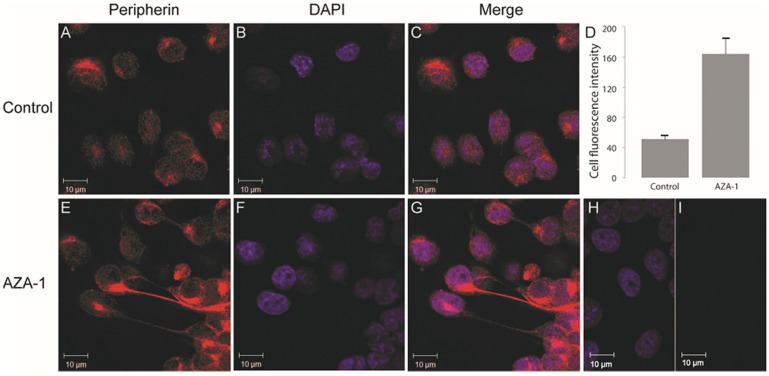
Neurite-like protrusions are strongly stained with peripherin. AZA-1 exposed (15 nM, 24 h) PC12 cells (**E**-**I**) and controls (**A**-**C**) were processed for immuno-labelling using anti-peripherin (red). Panel D shows relative mean cell fluorescence ± sem (*n* = 30). Panel **H**/**I** show negative control with no primary antibody added. Nuclei were stained with DAPI (blue).

Since AZA-1-exposed cells appeared more differentiated compared to control cells, we elucidated the expression of the PC12-associated neuronal differentiation marker, peripherin [26]. In untreated cells, peripherin was observed in patches of the whole cell with little specific organisation or filament structures (Figure 3A–C). In cells exposed to AZA-1, neurite-like protrusions with condensed bundles of peripherin filaments were frequently observed (Figure 3E–G) and mean cell fluorescence was increased compared to control (Figure 3D). Furthermore, AZA-1-exposed cells showed more condensed labelling of peripherin in close proximity to the nucleus.

The peripherin filaments observed in the protrusions further support the possibility that AZA-1 triggers a differentiation process, as peripherin is known to label neurites in differentiated PC12 cells [27].

**Figure 4:**
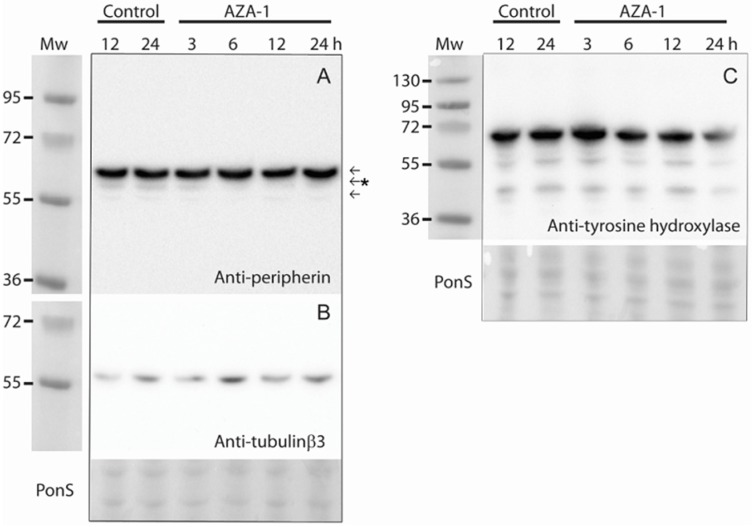
Time-dependent effect of AZA-1 on the neuronal differentiation markers peripherin, tubulinβ3, and tyrosine hydroxylase. Cells were exposed to 15 nM AZA-1 or negative control for up to 24 h and differentiation marker expression was analysed by Western blotting. (**A**) Three peripherin bands were observed whereas the second band (indicated by an asterisk) was down-regulated at all examined time points after addition of AZA-1; (**B**) The blot was then stripped and reprobed with anti-tubulinβ3; (**C**) Tyrosine hydroxylase expression. PonS stainings (**A**,**B**) and (**C**) are shown as loading control.

To observe possible changes in peripherin isoform levels after AZA-1 exposure cell lysates were analysed by Western blotting. The peripherin antibody used recognised three different bands, ranging from 50 to 60 kDa (Figure 4A). This is in accordance with Robertson *et al*. [28] who used the same antibody.

The differentiation-inducing effect of AZA-1 was also tested by analysing the effect on the neuronal markers tyrosine hydroxylase and tubulinβ3 [29]. Tyrosine hydroxylase showed an early transient increase after AZA- exposure (Figure 4C), whilst little effect was observed on tubulinβ3 (Figure 4B).

**Figure 5:**
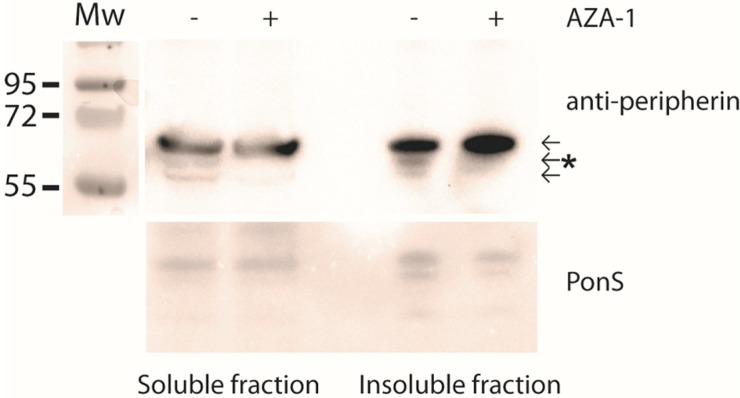
The down-regulated peripherin isoform is not found in the insoluble fraction. Cells were treated with 15 nM AZA-1or solvent control for 24 h and lysed in homogenisation buffer. The insoluble pellet obtained after centrifugation was dissolved in SDS-containing sample buffer. Western blot analysis with peripherin antibody showed that the second peripherin band disappeared both in the soluble and insoluble fraction after AZA-1 treatment. PonS staining was used as loading control. Molecular weight in kDa.

It is well known that intermediate filaments are not always soluble in CHAPS detergent, which was used in the lysis buffer [30]. To ensure that the down-regulated peripherin isoform did not remain in the CHAPS-insoluble fraction, the pellet was also analysed by Western blotting (Figure 5). As shown in Figure 5, the down-regulated peripherin isoform did not appear in the insoluble pellet.

### 2.3. Mass Spectrometry-Based Verification of Peripherin Isoforms

**Figure 6:**
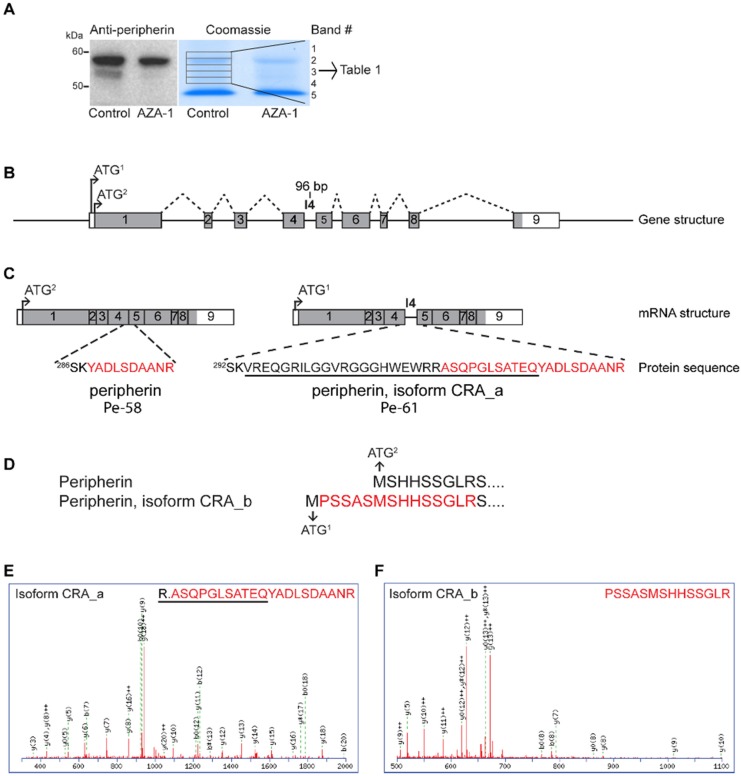
Identification of peripherin isoforms by mass spectrometry. (**A**) Proteins from total cell lysates (15 nM AZA-1 or negative control) were separated by SDS-PAGE. The gels were either further processed for analysis by Western blotting using a peripherin antibody or stained with Coomassie. The Western blot was used as guidance for excision of five gel slices (from the control lysate) of the Coomassie-stained gel. The proteins in the gel slices were in-gel digested and analysed by mass spectrometry. Peripherin isoforms were identified in all five gel slices (see Table 1). (**B**) Structure of the rat peripherin gene (ENSRNOG00000015643; 1 September 2014). The gene consists of 9 exons, labelled 1–9. Grey areas indicate protein-coding sequences. There are two start codons in-frame with the longest open reading frame, of which the second (ATG^2^) is considered the canonical start codon. Normally all introns are spliced out to give rise to peripherin. Dotted black lines indicates a possible alternative splicing event in which intron 4 (**I4**) is retained, giving rise to a 96-bp-long insert between exon 4 and exon 5 in the mature mRNA. This splicing event has previously been shown to take place in mouse peripherin mRNA and is named Pe-61. (**C**) The two possible mRNAs resulting from the splicing events described in (**B**). Retention of I4 gives rise to a 32 amino acid long insert (underlined in black in the protein sequence). A tryptic peptide unique for the peripherin isoform CRA_a/Pe-61 which is identified by mass spectrometry is labelled in red. As can be seen, parts of intron 4 connected to exon 5 was identified with the mass spectrometer, indicating that this isoform of peripherin does exist in rats. Note that isoform CRA_a/Pe-61 uses the first in-frame start codon, resulting in 6 additional amino acids on its N-terminal and hence a different numbering of the amino acids. (**D**) The N-terminal amino acid sequence of peripherin and the predicted peripherin isoform CRA_b, in which the first in-frame start codon is used. Our mass spectrometry data identified a tryptic peptide containing five of the six most N-terminal amino acids in peripherin isoform CRA_b. (**E**) Mass spectrum of the tryptic peptide consisting of parts of intron 4 connected to exon 5 in peripherin isoform CRA_a/Pe-61. (**F**) Mass spectrum of the N-terminal tryptic peptide in peripherin isoform CRA_b.

To confirm that the down-regulated band observed by Western blotting was peripherin, mass spectrometry was utilised. Proteins were separated by SDS-PAGE and bands corresponding to peripherin were excised and analysed by mass spectrometry (Figure 6A). Peripherin was identified in all five gel slices (Table 1), further supporting that the down-regulated band was peripherin.

**Table 1:** Peripherin isoforms identified in gel bands.

GI	Protein Name	No of Amino Acids	Band ^#^				
			1	2	3	4	5
149032092	Peripherin, isoform CRA_a	506	x				
129822	Peripherin	468	x		x	x	X
149032093	Peripherin, isoform CRA_b	474		x			

GI: GenInfo identifier. ^#^: Gel band number as in Figure 6A.

In addition to identifying the known main variant of rat peripherin protein (GI: 129822; hereafter called Pe-58 in accordance with the naming of peripherin isoforms in mouse [31]), previously predicted peripherin isoforms were also found (Figure 6B–F, Table 1, Supplementary Materials Tables S1–S5).

Particularly interesting was the identification of peripherin isoform CRA_a (GI: 149032092). This isoform was found exclusively in band #1 (Figure 6A,E). In this isoform, intron four is retained, giving rise to a 32-amino-acid-long insert in the protein sequence and hence an increased mass compared to Pe-58 (Figure 6C). This alternatively spliced isoform is known in mouse as Pe-61 [31,32]; however, to our knowledge, it has not been previously identified in rat. Additionally, we identified CRA_b (GI: 149032093) in band #2 (Figure 6A,F). In this isoform the first in-frame start codon is used, giving rise to six additional N-terminal amino acids (Figure 6D).

The question remains as to which isoform is down-regulated by AZA-1 exposure. It is tempting to assume that the most intense band on the Western blot (Figure 4A and Figure 6A) represents the Pe-58 isoform as it runs as a ~58 kDa protein, which is in good accordance with previous reports [31,32]. In addition, Pe-58 is the constitutively expressed isoform, making it reasonable that it represents the most intense band. This would imply that the identified isoform CRA_a/Pe-61 is not detected with the antibody used in this study, possibly due to amounts below the detection limit [28]. In mice, a third peripherin isoform, which runs on SDS-polyacrylamide gels as a 56 kDa protein, has been described [31,32]. This would fit well with the observed molecular weight of the down-regulated peripherin isoform in our study.

### 2.4. Peripherin is Not Down-Regulated at the Transcriptional Level

We then tested whether AZA-1-induced peripherin down-regulation could be assigned to the transcriptional level. PC12 cells were treated with 15 nM AZA-1 for 24 or 48 h or with negative control, and reverse transcriptase-PCR was used to examine relative expression levels. The forward primer located at the exon four–exon five junction and reverse primer located in exon nine were used to detect peripherin. This primer pair would recognise both Pe-58 and a potential Pe-56 variant if it exists in rats (the Pe-61 variant would not be detected using this primer pair, but we had already excluded it as being the down-regulated band due to its larger size). One band at the expected size for Pe-58 was detected, but no regulation of the expression of peripherin mRNA was observed (data not shown).

### 2.5. Peripherin Down-Regulation Is Not Dependent on the Proteasome, Caspases and Calpains

**Figure 7:**
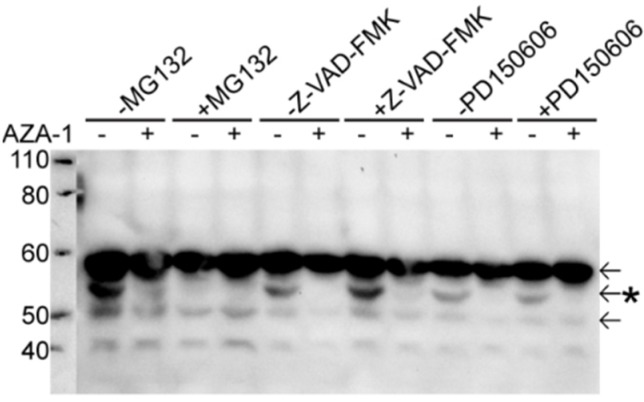
The proteasome, caspases and calpains are not involved in peripherin down-regulation after AZA-1 exposure. Cells were pre-incubated for 1 h with a proteasome inhibitor (MG132), caspase inhibitor (Z-VAD-FMK) or calpain inhibitor (PD150606) prior to exposure to 15 nM AZA-1 or negative control for 24 h. Expression of peripherin isoforms (arrows) was detected using Western blotting. Pre-incubation with the different inhibitors did not prevent down-regulation of the second peripherin band (indicated by an asterisk). It was noted that pre-treatment with the proteasome inhibitor MG132 alone also led to a down-regulation of the second peripherin band (third lane from left).

The peripherin isoform is completely disappeared within three hours after AZA-1 administration. Since the half-life of peripherin is 33 h [27], this rapid and complete down-regulation indicates a degradation/cleavage of the protein rather than a down-regulation of the mRNA. We therefore tested if the down-regulated peripherin isoform was degraded by the proteasome system. However, inhibition of the proteasome did not prevent the observed disappearance of the peripherin isoform after AZA-1 treatment (Figure 7). Interestingly, pre-incubation with the proteasome inhibitor by itself also led to disappearance of the peripherin isoform, even in the absence of AZA-1 treatment. Furthermore, it was noted that the morphology of MG132-treated cells resembled the morphology of AZA-1-treated cells, with neurite-like protrusions occurring (data not shown). Since AZA-1 and MG132 produced similar effects, we checked if AZA-1 acted as a proteasome inhibitor by assessing the degree of protein ubiquitination after AZA-1 exposure. Pre-incubation with MG132 increased the overall levels of ubiquitination as expected, but AZA-1 treatment did not result in any accumulation of protein ubiquitination (data not shown), indicating that AZA-1 did not inhibit the proteasome.

We examined if peripherin was degraded by apoptosis-associated caspases as it has previously been shown that AZA-1 activated caspases in a number of different cell types [8,11]. However, pre-incubation with the broad caspase inhibitor Z-VAD-FMK did not prevent down-regulation of the peripherin isoform after AZA-1 treatment (Figure 7).

Kim *et al.* have recently identified peripherin as a novel calpain target [33]. Furthermore, calpains are Ca^2+^-dependent [34] and AZA-1 treatment has been shown to elevate intracellular calcium levels [10], but no calpain inhibitor effect was observed (Figure 7).

## 3. Discussion

Exposure to the algal toxin AZA-1 has shown to induce neurotoxicological symptoms [1,4], and a number of studies have been performed on neuronal cells [10,11,12] and their derivatives [7,8,9], but its biological target is still not known. In this study we have used PC12 cells. This cell line is commonly used as a model for the enteric nervous system [19], by which oral exposure to the toxin will be the first part of the neuronal system to be reached. We here show that AZA-1 down-regulates a specific isoform of the intermediate filament protein peripherin in PC12 cells. Although long-term exposure to AZA-1 decreased cell viability, a more differentiated phenotype was observed in the early stage after toxin addition. The differentiated cellular phenotype with neurite-like protrusions was observed simultaneously with the change in peripherin isoform stoichiometry.

Peripherin is mainly expressed in the peripheral nervous system [25,35] and found in pathological inclusions of patients with amyotrophic lateral sclerosis (ALS) [36]. It has also been shown that peripherin isoform expression profiles are altered in ALS and after neuronal injury [37]. The functional relevance of the different isoforms is not known, but when expressed in SW13 vim (−), a cell line lacking cytoplasmic intermediate filament proteins, distinct morphologies and filament assembly are observed [28,38]. Transgenic mice over-expressing peripherin show selective motor neuron degeneration [39]. On the other hand, up-regulation of peripherin is observed after neuronal injury [37,40] and has been shown to be cytoprotective [41], further supporting that specific peripherin isoform expression may have individual roles.

Although we cannot be sure of the identity of the down-regulated peripherin isoform, it is tempting to believe that it is homologous to the mouse Pe-56 isoform [31,32]. This fits well with the molecular weight observed and expression level compared to the main constitutively expressed isoform Pe-58. Pe-56 has been shown to be down-regulated in response to a low level of oxidative stress in mouse neuroblastoma cells, although at higher levels of oxidative stress Pe-56 levels remained at control level [42]. In our hands, AZA-1 did not induce oxidative stress as judged by oxyblotting towards protein carbonylation (data not shown). This is in accordance with Vale *et al.* [10], who showed that AZA-1 did not increase oxidative stress using a reactive oxygen species sensitive fluorescent probe. Thus, the down-regulation of Pe-56 may not necessarily be directly coupled to oxidative stress.

We observed a concomitant down-regulation of a specific peripherin isoform and increased cell differentiation with peripherin filament assembly in neurite-like protrusions following AZA-1 exposure of PC12 cells. Previous studies have shown peripherin expression to be necessary for PC12 cells to form neuritic processes upon differentiation induced by nerve growth factor [27]. Overall, this indicates that the presence of Pe-56 is not essential in the developing neurites.

We checked if peripherin was degraded by caspases or calpains, both involved in apoptosis, but peripherin isoform down-regulation does not appear to be mediated through these proteases. A possible degradation of peripherin isoform by the proteasome system was explored by using proteasome inhibitor MG132. Down-regulation of peripherin isoform was not prevented, but we observed that MG132 and AZA-1 both induced down-regulation of the same peripherin isoform and a similar differentiated morphological appearance [43]. Also, in a recent study on the effect of the proteasome inhibitor lactacystin on PC12 cells, peripherin was identified to be down-regulated by this proteasome inhibitor [44]. However, the authors did not show which isoform is down-regulated or what the mechanism behind this down-regulation might be. It is rather puzzling that proteasome inhibition would lead to peripherin down-regulation. However, MG132 has been shown to have several targets in the cells in addition to being a proteasome inhibitor [43,45,46,47].

Dysregulation of peripherin and its splice variants has been associated with the neurodegenerative disease ALS [28,37,38,48]. Therefore, the fact that an algal toxin specifically down-regulates a peripherin isoform may provide a new tool for understanding the relevance of peripherin in neuronal cell death.

In conclusion, we have shown that exposure to AZA-1 induces an early differentiated phenotype followed by later cell death in PC12 cells. The differentiated appearance coincides with down-regulation of a specific peripherin isoform, a neuronal specific intermediate filament protein.

## 4. Experimental Section

All chemicals were from Sigma-Aldrich unless otherwise stated.

### 4.1. Cell Culturing and Treatment

Rat adrenal pheochromocytoma PC12 cells were cultured as previously described in [49]. Cells were seeded to obtain a confluency of around 70% at the time of exposure to 15 nM AZA-1 (cat. No. CRM-AZA1, National Research Council, ON, Canada) or solvent control (DMSO). The following inhibitors were used: 1 μM proteasome inhibitor MG132 (#C2211, Sigma-Aldrich, St. Louis, MO, USA),; 100 μM caspase inhibitor Z-VAD-FMK (#FMK001, R&D Systems, Minneapolis, MN, USA); 100 μM calpain inhibitor PD150606 (#D5946, Sigma-Aldrich, St. Louis, MO, USA).

### 4.2. Evaluation of Cell Morphology and Viability

Measurement of neurite-like outgrowth was performed on micrographs taken of live cells by manually measuring outgrowth length compared to perikaryon diameter. Outgrowths were counted as “neurite-like” if outgrowth lengths were more than 0.5 times of perikaryon diameter. At least 130 cells from three fields per well were evaluated.

Cell viability was measured using Cell Counting Kit-8 (CCK-8)( Sigma-Aldrich, St. Louis, MO, USA) according to the manufacturer’s protocol. PC12 cells were seeded in 96-well plates (30,000 cells/well) 24 h before treatment with or without AZA-1. Attached cells were trypsinated and counted together with detached cells using a Neubauer chamber.

### 4.3. Immunofluorescence

Cells (220,000 cells/well) were seeded on poly-l-Lysine coated cover slips in a 24-well plate one day prior to exposure to 15 nM AZA-1 or solvent control for 24 h. Cells were prepared for immunofluorescence staining as previously described [9] using anti-peripherin (1:400, AB1530, Millipore, Billerica, MA, USA) and AlexaFluor594 anti-rabbit (1:200A11012, ThermoFisher, Waltham, MA, USA). Nuclei were stained using DAPI. Images were captured with a Zeiss LSM 510 META confocal microscope (Zeiss, Oberkochen, Germany). Cell fluorescence was quantitated using ImageJ version 1.47 according to the formula: Corrected total cell fluorescence = Integrated density—(area of selected cell x mean fluorescence of background readings).

### 4.4. SDS-PAGE and Western Blotting

Cells were lysed in homogenisation buffer (10 mM K_2_HPO_4_, 10 mM KH_2_PO_4_, 1 mM EDTA, 0.6% (*w*/*v*) 3-((3-cholamidopropyl)dimethylammonio)-1-propanesulfonate (CHAPS), 0.2 mM Na_3_VO_4_, 50 mM NaF, Complete Protease Inhibitor Cocktail (Roche Diagnostics, Mannheim, Germany)and cleared by centrifugation. For analysis of the insoluble fraction, the pellet after centrifugation was dissolved in 100 μL SDS sample buffer and 20 μL loaded. Proteins separated on 8% SDS-polyacrylamide gels were either stained with Coomassie or transferred to PDVF membranes (RPN303F, GE Healthcare, Buckinghamshire, UK). Membranes were blocked in 1% BSA in PBS-Tween for one hour. Membranes were incubated with anti-peripherin rabbit polyclonal antibody (Millipore, Billerica, MA, USA AB1530, 1:1000), anti-betaTubulin3 mouse monoclonal antibody (Santa Cruz, Dallas, TX USA sc-51670, 1:500), or anti-tyrosine hydroxylase mouse monoclonal antibody (SigmaAldrich, St. Louis, MO, USA, T2928, 1:1000) for one h at RT, followed by peroxidase-conjugated anti-rabbit or anti-mouse antibodies (Jackson ImmunoResearch, West Baltimore Pike, West Grove,PA, USA, 1:10,000, 1 h at RT). Blots were developed with one mL ECL solution (ThermoFisher, Waltham, MA, USA) using a ChemiDoc™ (Bio-Rad, Hercules, CA, USA). Washing with PBS-T (3 × 10 min) was performed between all incubation steps. In all gels used for Western blotting, 40 μg protein was loaded per well. Ponceau-S staining was used as loading control.

### 4.5. RNA Isolation and Reverse Transcription

Total RNA from PC12 cells were extracted with Trizol (ThermoScientific, Waltham, MA, USA) according to the manufacture instruction. RNA concentration and quality was assed using NanoDrop ND1000 (ThermoScientific, Waltham, MA, USA). To synthesize cDNA, Oligo dT primer and Transcriptor reverse transcriptase (Roche, Basel, Switzerland) was used. To detect the transcriptional level of Pe-58 and Pe-56, 500 ng cDNA was amplified by PCR using forward primer 5′-AAGAAGCTACACGAAGAGGAG-3′ and reverse primer 5′-GGAGGGTTCGAGCTTAGGAA-3′.

### 4.6. Identification of Peripherin Isoforms

A Western blot was compared to a Coomassie-stained protein gel and bands were excised manually from the gel. Proteins were in-gel digested with trypsin (V5111, Promega, Madison, WI, USA) and peptides were extracted and reduced/alkylated according to Kellmann *et al.* [9]. Mass spectrometry analysis was performed according to [50], with the following modifications: the 15 ions with highest intensity were sequenced, normalized collision energy was 35%, dynamic exclusion was 30 s.

### 4.7. Data Analysis

Raw data were converted to mgf files with Proteome discoverer™ software version 1.3 (ThermoScientific, Waltham, MA, USA) and searched against NCBInr (2012.04.15; taxonomy *Rattus,* 67,662 sequences) using Mascot Daemon version 2.3.0 (Matrix Science, Boston, MA, USA) and the Mascot search engine (http://www.matrixscience.com). Fixed modification was carbamidomethylated cysteines, whereas variable modifications were oxidised methionine, nitrosylated tyrosine and phosphorylated serine, threonine and tyrosine. Mass tolerance for MS and MS/MS was set to 10 ppm and 0.6 Da respectively, and maximum allowed missed cleavages were two. Proteins were identified with a significant threshold of *p* < 0.05 at peptide level.

## Supplementary Materials

See Supplementary Materials Tables S1–S5.

## Abbreviations

ALS	amyotrophic lateral sclerosis
AZA-1	azaspiracid-1
CCK-8	Cell Counting Kit-8
DSP	diarrhetic shellfish poisoning
