# Supplementary material for: Algal Toxin Azaspiracid-1 Induces Early Neuronal Differentiation and Alters Peripherin Isoform Stoichiometry
Source: Mar Drugs. 2015 Dec 14;13(12):7390–402. doi: 10.3390/md13127072 (PMC4699245; doi:10.3390/md13127072)
Supplement: Supplementary file 1 [file marinedrugs-13-07072-s001.docx]

Supplementary Materials: The Algal Toxin Azaspiracid-1 Induces an Early Neuronal Differentiation and Alters Peripherin Isoform Stoichiometry

Linda V. Hjørnevik, Ann K. Frøyset, Toril A. Grønset, Krisna Rungruangsak-Torrissen and Kari E. Fladmark

**Table S1.** Identified tryptic peptides in sample 1.

| gi\|149032092 peripherin 1, isoform CRA_a [*Rattus norvegicus*] | | | | | | |
| --- | --- | --- | --- | --- | --- | --- |
| **Start–End** | **Observed** | **Mr (expt)** | **Mr (calc)** | **ppm** | **Miss** | **Sequence** |
| 26–45 | 676.9954 | 2027.9643 | 2027.9589 | 3 | 0 | R.TFGPPPSLSPGAFSYSSSSR.F (Ions score 24) |
| 26–45 | 1014.9897 | 2027.9649 | 2027.9589 | 3 | 0 | R.TFGPPPSLSPGAFSYSSSSR.F (Ions score 66) |
| 26–45 | 1014.99 | 2027.9654 | 2027.9589 | 3 | 0 | R.TFGPPPSLSPGAFSYSSSSR.F (Ions score 81) |
| 51–62 | 599.7783 | 1197.5421 | 1197.5391 | 3 | 0 | R.LLGSGSPSSSAR.L Phospho (ST) (Ions score 23) |
| 71–80 | 535.3108 | 1068.607 | 1068.604 | 3 | 1 | R.AGALRLPSER.L (Ions score 21) |
| 71–80 | 357.2097 | 1068.6072 | 1068.604 | 3 | 1 | R.AGALRLPSER.L (Ions score 23) |
| 98–110 | 801.8919 | 1601.7693 | 1601.7645 | 3 | 1 | R.SNEKQELQELNDR.F (Ions score 75) |
| 98–110 | 534.9304 | 1601.7694 | 1601.7645 | 3 | 1 | R.SNEKQELQELNDR.F (Ions score 50) |
| 111–117 | 434.7328 | 867.4511 | 867.449 | 2 | 0 | R.FANFIEK.V (Ions score 37) |
| 111–117 | 434.733 | 867.4515 | 867.449 | 3 | 0 | R.FANFIEK.V (Ions score 16) |
| 120–129 | 595.3218 | 1188.6291 | 1188.6251 | 3 | 0 | R.FLEQQNAALR.G (Ions score 57) |
| 120–136 | 644.3434 | 1930.0083 | 1930.002 | 3 | 1 | R.FLEQQNAALRGELSQAR.G (Ions score 45) |
| 143–152 | 630.8054 | 1259.5963 | 1259.5928 | 3 | 0 | R.ADQLCQQELR.E (Ions score 41) |
| 156–163 | 493.2941 | 984.5736 | 984.5716 | 2 | 1 | R.RELELLGR.E (Ions score 22) |
| 156–165 | 635.8643 | 1269.714 | 1269.7153 | −1 | 2 | R.RELELLGRER.D (Ions score 1) |
| 157–163 | 415.2432 | 828.4719 | 828.4705 | 2 | 0 | R.ELELLGR.E (Ions score 42) |
| 173–183 | 551.2944 | 1100.5742 | 1100.5713 | 3 | 0 | R.DGLAEDLGALK.Q (Ions score 46) |
| 173–183 | 551.2952 | 1100.5758 | 1100.5713 | 4 | 0 | R.DGLAEDLGALK.Q (Ions score 42) |
| 173–185 | 462.5856 | 1384.735 | 1384.731 | 3 | 1 | R.DGLAEDLGALKQR.L (Ions score 9) |

**Table S1.** *Cont.*

| 192–204 | 542.9634 | 1625.8685 | 1625.8638 | 3 | 2 | R.KREDAEHNLVLFR.K (Ions score 25) |
| --- | --- | --- | --- | --- | --- | --- |
| 192–204 | 407.4745 | 1625.8689 | 1625.8638 | 3 | 2 | R.KREDAEHNLVLFR.K (Ions score 34) |
| 192–204 | 813.9417 | 1625.8689 | 1625.8638 | 3 | 2 | R.KREDAEHNLVLFR.K (Ions score 62) |
| 193–204 | 500.2645 | 1497.7716 | 1497.7688 | 2 | 1 | K.REDAEHNLVLFR.K (Ions score 27) |
| 194–204 | 448.23 | 1341.6683 | 1341.6677 | 0 | 0 | R.EDAEHNLVLFR.K (Ions score 5) |
| 194–204 | 448.2317 | 1341.6734 | 1341.6677 | 4 | 0 | R.EDAEHNLVLFR.K (Ions score 7) |
| 205–219 | 587.3132 | 1758.9177 | 1758.9112 | 4 | 2 | R.KDVDDATLSRLELER.K (Ions score 8) |
| 206–219 | 544.6138 | 1630.8195 | 1630.8162 | 2 | 1 | K.DVDDATLSRLELER.K (Ions score 26) |
| 220–233 | 580.3162 | 1737.9268 | 1737.9222 | 3 | 2 | R.KIESLMDEIEFLKK.L Oxidation (M) (Ions score 39) |
| 273–281 | 504.2629 | 1006.5113 | 1006.5083 | 3 | 0 | R.AQYENIAAK.N (Ions score 24) |
| 282–291 | 655.3082 | 1308.6018 | 1308.5986 | 2 | 0 | K.NLQEAEEWYK.S (Ions score 42) |
| 282–295 | 952.9391 | 1903.8636 | 1903.8465 | 9 | 2 | K.NLQEAEEWYKSKVR.E Nitro (Y); Phospho (ST) (Ions score 0) |
| 315–335 | 1083.011 | 2164.0074 | 2164.0032 | 2 | 0 | R.ASQPGLSATEQYADLSDAANR.N (Ions score 100) |
| 315–335 | 1083.0144 | 2164.0142 | 2164.0032 | 5 | 0 | R.ASQPGLSATEQYADLSDAANR.N (Ions score 107) |
| 352–365 | 558.9593 | 1673.856 | 1673.8519 | 2 | 1 | R.RQIQSLTCEVDGLR.G (Ions score 25) |
| 353–365 | 759.8845 | 1517.7544 | 1517.7508 | 2 | 0 | R.QIQSLTCEVDGLR.G (Ions score 51) |
| 413–421 | 568.3051 | 1134.5955 | 1134.5921 | 3 | 0 | R.EYQELLNVK.M (Ions score 39) |
| 413–421 | 590.7916 | 1179.5686 | 1179.5771 | −7 | 0 | R.EYQELLNVK.M Nitro (Y) (Ions score 7) |
| 442–455 | 495.6216 | 1483.8429 | 1483.8399 | 2 | 0 | R.ISVPVHSFASLSLK.T (Ions score 17) |
| 456–470 | 560.2739 | 1677.7998 | 1677.7958 | 2 | 0 | K.TTVPEVEPPQDSHSR.K (Ions score 10) |
| 456–470 | 839.9081 | 1677.8017 | 1677.7958 | 4 | 0 | K.TTVPEVEPPQDSHSR.K (Ions score 53) |
| 456–471 | 452.4817 | 1805.8979 | 1805.8908 | 4 | 1 | K.TTVPEVEPPQDSHSRK.M (Ions score 18) |
| 456–471 | 602.9734 | 1805.8985 | 1805.8908 | 4 | 1 | K.TTVPEVEPPQDSHSRK.M (Ions score 16) |
| 471–476 | 380.25 | 758.4854 | 758.4836 | 2 | 1 | R.KMVLIR.T (Ions score 37) |
| 471–476 | 388.2478 | 774.481 | 774.4786 | 3 | 1 | R.KMVLIR.T Oxidation (M) (Ions score 28) |
| 472–476 | 316.2024 | 630.3903 | 630.3887 | 3 | 0 | K.MVLIR.T (Ions score 24) |
| 486–500 | 586.2976 | 1755.8708 | 1755.8639 | 4 | 1 | K.VVTESQKEQHSELDK.S (Ions score 13) |
| 486–506 | 608.551 | 2430.1747 | 2430.1663 | 3 | 2 | K.VVTESQKEQHSELDKSSIHSY—(Ions score 17) |
| 486–506 | 811.0656 | 2430.1748 | 2430.1663 | 4 | 2 | K.VVTESQKEQHSELDKSSIHSY—(Ions score 35) |
| 493–506 | 553.9271 | 1658.7595 | 1658.7536 | 4 | 1 | K.EQHSELDKSSIHSY—(Ions score 27) |

**Table S1.** *Cont.*

| gi\|129822 RecName: Full=Peripherin | | | | | | |
| --- | --- | --- | --- | --- | --- | --- |
| Proteins matching the same set of peptides | | | | | | |
| gi\|149032093 peripherin 1, isoform CRA_b | | | | | | |
| **Start–End** | **Observed** | **Mr (expt)** | **Mr (calc)** | **ppm** | **Miss** | **Sequence** |
| 20–39 | 676.9954 | 2027.9643 | 2027.9589 | 3 | 0 | R.TFGPPPSLSPGAFSYSSSSR.F (Ions score 24) |
| 20–39 | 1014.9897 | 2027.9649 | 2027.9589 | 3 | 0 | R.TFGPPPSLSPGAFSYSSSSR.F (Ions score 66) |
| 20–39 | 1014.99 | 2027.9654 | 2027.9589 | 3 | 0 | R.TFGPPPSLSPGAFSYSSSSR.F (Ions score 81) |
| 45–56 | 599.7783 | 1197.5421 | 1197.5391 | 3 | 0 | R.LLGSGSPSSSAR.L Phospho (ST) (Ions score 23) |
| 65–74 | 535.3108 | 1068.607 | 1068.604 | 3 | 1 | R.AGALRLPSER.L (Ions score 21) |
| 65–74 | 357.2097 | 1068.6072 | 1068.604 | 3 | 1 | R.AGALRLPSER.L (Ions score 23) |
| 92–104 | 801.8919 | 1601.7693 | 1601.7645 | 3 | 1 | R.SNEKQELQELNDR.F (Ions score 75) |
| 92–104 | 534.9304 | 1601.7694 | 1601.7645 | 3 | 1 | R.SNEKQELQELNDR.F (Ions score 50) |
| 105–111 | 434.7328 | 867.4511 | 867.449 | 2 | 0 | R.FANFIEK.V (Ions score 37) |
| 105–111 | 434.733 | 867.4515 | 867.449 | 3 | 0 | R.FANFIEK.V (Ions score 16) |
| 114–123 | 595.3218 | 1188.6291 | 1188.6251 | 3 | 0 | R.FLEQQNAALR.G (Ions score 57) |
| 114–130 | 644.3434 | 1930.0083 | 1930.002 | 3 | 1 | R.FLEQQNAALRGELSQAR.G (Ions score 45) |
| 137–146 | 630.8054 | 1259.5963 | 1259.5928 | 3 | 0 | R.ADQLCQQELR.E (Ions score 41) |
| 150–157 | 493.2941 | 984.5736 | 984.5716 | 2 | 1 | R.RELELLGR.E (Ions score 22) |
| 150–159 | 635.8643 | 1269.714 | 1269.7153 | −1 | 2 | R.RELELLGRER.D (Ions score 1) |
| 151–157 | 415.2432 | 828.4719 | 828.4705 | 2 | 0 | R.ELELLGR.E (Ions score 42) |
| 167–177 | 551.2944 | 1100.5742 | 1100.5713 | 3 | 0 | R.DGLAEDLGALK.Q (Ions score 46) |
| 167–177 | 551.2952 | 1100.5758 | 1100.5713 | 4 | 0 | R.DGLAEDLGALK.Q (Ions score 42) |
| 167–179 | 462.5856 | 1384.735 | 1384.731 | 3 | 1 | R.DGLAEDLGALKQR.L (Ions score 9) |
| 186–198 | 542.9634 | 1625.8685 | 1625.8638 | 3 | 2 | R.KREDAEHNLVLFR.K (Ions score 25) |
| 186–198 | 407.4745 | 1625.8689 | 1625.8638 | 3 | 2 | R.KREDAEHNLVLFR.K (Ions score 34) |
| 186–198 | 813.9417 | 1625.8689 | 1625.8638 | 3 | 2 | R.KREDAEHNLVLFR.K (Ions score 62) |
| 187–198 | 500.2645 | 1497.7716 | 1497.7688 | 2 | 1 | K.REDAEHNLVLFR.K (Ions score 27) |
| 188–198 | 448.230 | 1341.6683 | 1341.6677 | 0 | 0 | R.EDAEHNLVLFR.K (Ions score 5) |
| 188–198 | 448.2317 | 1341.6734 | 1341.6677 | 4 | 0 | R.EDAEHNLVLFR.K (Ions score 7) |

**Table S1.** *Cont.*

| 199–213 | 587.3132 | 1758.9177 | 1758.9112 | 4 | 2 | R.KDVDDATLSRLELER.K (Ions score 8) |
| --- | --- | --- | --- | --- | --- | --- |
| 200–213 | 544.6138 | 1630.8195 | 1630.8162 | 2 | 1 | K.DVDDATLSRLELER.K (Ions score 26) |
| 214–227 | 580.3162 | 1737.9268 | 1737.9222 | 3 | 2 | R.KIESLMDEIEFLKK.L Oxidation (M) (Ions score 39) |
| 267–275 | 504.2629 | 1006.5113 | 1006.5083 | 3 | 0 | R.AQYENIAAK.N (Ions score 24) |
| 276–285 | 655.3082 | 1308.6018 | 1308.5986 | 2 | 0 | K.NLQEAEEWYK.S (Ions score 42) |
| 288–297 | 548.2586 | 1094.5027 | 1094.4992 | 3 | 0 | K.YADLSDAANR.N (Ions score 51) |
| 288–303 | 454.7247 | 1814.8698 | 1814.8659 | 2 | 1 | K.YADLSDAANRNHEALR.Q (Ions score 22) |
| 288–303 | 605.9647 | 1814.8722 | 1814.8659 | 3 | 1 | K.YADLSDAANRNHEALR.Q (Ions score 32) |
| 288–303 | 908.4434 | 1814.8722 | 1814.8659 | 3 | 1 | K.YADLSDAANRNHEALR.Q (Ions score 19) |
| 314–327 | 558.9593 | 1673.856 | 1673.8519 | 2 | 1 | R.RQIQSLTCEVDGLR.G (Ions score 25) |
| 315–327 | 759.8845 | 1517.7544 | 1517.7508 | 2 | 0 | R.QIQSLTCEVDGLR.G (Ions score 51) |
| 375–383 | 568.3051 | 1134.5955 | 1134.5921 | 3 | 0 | R.EYQELLNVK.M (Ions score 39) |
| 375–383 | 590.7916 | 1179.5686 | 1179.5771 | −7 | 0 | R.EYQELLNVK.M Nitro (Y) (Ions score 7) |
| 404–417 | 495.6216 | 1483.8429 | 1483.8399 | 2 | 0 | R.ISVPVHSFASLSLK.T (Ions score 17) |
| 418–432 | 560.2739 | 1677.7998 | 1677.7958 | 2 | 0 | K.TTVPEVEPPQDSHSR.K (Ions score 10) |
| 418–432 | 839.9081 | 1677.8017 | 1677.7958 | 4 | 0 | K.TTVPEVEPPQDSHSR.K (Ions score 53) |
| 418–433 | 452.4817 | 1805.8979 | 1805.8908 | 4 | 1 | K.TTVPEVEPPQDSHSRK.M (Ions score 18) |
| 418–433 | 602.9734 | 1805.8985 | 1805.8908 | 4 | 1 | K.TTVPEVEPPQDSHSRK.M (Ions score 16) |
| 433–438 | 380.25 | 758.4854 | 758.4836 | 2 | 1 | R.KMVLIR.T (Ions score 37) |
| 433–438 | 388.2478 | 774.481 | 774.4786 | 3 | 1 | R.KMVLIR.T Oxidation (M) (Ions score 28) |
| 434–438 | 316.2024 | 630.3903 | 630.3887 | 3 | 0 | K.MVLIR.T (Ions score 24) |
| 448–462 | 586.2976 | 1755.8708 | 1755.8639 | 4 | 1 | K.VVTESQKEQHSELDK.S (Ions score 13) |
| 448–468 | 608.551 | 2430.1747 | 2430.1663 | 3 | 2 | K.VVTESQKEQHSELDKSSIHSY—(Ions score 17) |
| 448–468 | 811.0656 | 2430.1748 | 2430.1663 | 4 | 2 | K.VVTESQKEQHSELDKSSIHSY—(Ions score 35) |
| 455–468 | 553.9271 | 1658.7595 | 1658.7536 | 4 | 1 | K.EQHSELDKSSIHSY—(Ions score 27) |

**Table S1.** *Cont.*

| gi\|72679690 Peripherin [*Rattus norvegicus*] | | | | | | |
| --- | --- | --- | --- | --- | --- | --- |
| Proteins matching the same set of peptides | | | | | | |
| gi\|166063971 peripherin [*Rattus norvegicus*] | | | | | | |
| **Start–End** | **Observed** | **Mr (expt)** | **Mr (calc)** | **ppm** | **Miss** | **Sequence** |
| 20–39 | 676.9954 | 2027.9643 | 2027.9589 | 3 | 0 | R.TFGPPPSLSPGAFSYSSSSR.F (Ions score 24) |
| 20–39 | 1014.9897 | 2027.9649 | 2027.9589 | 3 | 0 | R.TFGPPPSLSPGAFSYSSSSR.F (Ions score 66) |
| 20–39 | 1014.99 | 2027.9654 | 2027.9589 | 3 | 0 | R.TFGPPPSLSPGAFSYSSSSR.F (Ions score 81) |
| 45–56 | 599.7783 | 1197.5421 | 1197.5391 | 3 | 0 | R.LLGSGSPSSSAR.L Phospho (ST) (Ions score 23) |
| 65–74 | 535.3108 | 1068.607 | 1068.604 | 3 | 1 | R.AGALRLPSER.L (Ions score 21) |
| 65–74 | 357.2097 | 1068.6072 | 1068.604 | 3 | 1 | R.AGALRLPSER.L (Ions score 23) |
| 92–104 | 801.8919 | 1601.7693 | 1601.7645 | 3 | 1 | R.SNEKQELQELNDR.F (Ions score 75) |
| 92–104 | 534.9304 | 1601.7694 | 1601.7645 | 3 | 1 | R.SNEKQELQELNDR.F (Ions score 50) |
| 105–111 | 434.7328 | 867.4511 | 867.449 | 2 | 0 | R.FANFIEK.V (Ions score 37) |
| 105–111 | 434.733 | 867.4515 | 867.449 | 3 | 0 | R.FANFIEK.V (Ions score 16) |
| 114–123 | 595.3218 | 1188.6291 | 1188.6251 | 3 | 0 | R.FLEQQNAALR.G (Ions score 57) |
| 114–130 | 644.3434 | 1930.0083 | 1930.002 | 3 | 1 | R.FLEQQNAALRGELSQAR.G (Ions score 45) |
| 137–146 | 630.8054 | 1259.5963 | 1259.5928 | 3 | 0 | R.ADQLCQQELR.E (Ions score 41) |
| 150–157 | 493.2941 | 984.5736 | 984.5716 | 2 | 1 | R.RELELLGR.E (Ions score 22) |
| 150–159 | 635.8643 | 1269.714 | 1269.7153 | −1 | 2 | R.RELELLGRER.D (Ions score 1) |
| 151–157 | 415.2432 | 828.4719 | 828.4705 | 2 | 0 | R.ELELLGR.E (Ions score 42) |
| 167–177 | 551.2944 | 1100.5742 | 1100.5713 | 3 | 0 | R.DGLAEDLGALK.Q (Ions score 46) |
| 167–177 | 551.2952 | 1100.5758 | 1100.5713 | 4 | 0 | R.DGLAEDLGALK.Q (Ions score 42) |
| 167–179 | 462.5856 | 1384.735 | 1384.731 | 3 | 1 | R.DGLAEDLGALKQR.L (Ions score 9) |
| 186–198 | 542.9634 | 1625.8685 | 1625.8638 | 3 | 2 | R.KREDAEHNLVLFR.K (Ions score 25) |
| 186–198 | 407.4745 | 1625.8689 | 1625.8638 | 3 | 2 | R.KREDAEHNLVLFR.K (Ions score 34) |
| 186–198 | 813.9417 | 1625.8689 | 1625.8638 | 3 | 2 | R.KREDAEHNLVLFR.K (Ions score 62) |
| 187–198 | 500.2645 | 1497.7716 | 1497.7688 | 2 | 1 | K.REDAEHNLVLFR.K (Ions score 27) |
| 188–198 | 448.23 | 1341.6683 | 1341.6677 | 0 | 0 | R.EDAEHNLVLFR.K (Ions score 5) |
| 188–198 | 448.2317 | 1341.6734 | 1341.6677 | 4 | 0 | R.EDAEHNLVLFR.K (Ions score 7) |
| 199–213 | 587.3132 | 1758.9177 | 1758.9112 | 4 | 2 | R.KDVDDATLSRLELER.K (Ions score 8) |

**Table S1.** *Cont.*

| 200–213 | 544.6138 | 1630.8195 | 1630.8162 | 2 | 1 | K.DVDDATLSRLELER.K (Ions score 26) | |
| --- | --- | --- | --- | --- | --- | --- | --- |
| 214–227 | 580.3162 | 1737.9268 | 1737.9222 | 3 | 2 | R.KIESLMDEIEFLKK.L Oxidation (M) (Ions score 39) | |
| 267–275 | 504.2629 | 1006.5113 | 1006.5083 | 3 | 0 | R.AQYENIAAK.N (Ions score 24) | |
| 276–285 | 655.3082 | 1308.6018 | 1308.5986 | 2 | 0 | K.NLQEAEEWYK.S (Ions score 42) | |
| 288–297 | 548.2586 | 1094.5027 | 1094.4992 | 3 | 0 | K.YADLSDAANR.N (Ions score 51) | |
| 288–303 | 454.7247 | 1814.8698 | 1814.8659 | 2 | 1 | K.YADLSDAANRNHEALR.Q (Ions score 22) | |
| 288–303 | 605.9647 | 1814.8722 | 1814.8659 | 3 | 1 | K.YADLSDAANRNHEALR.Q (Ions score 32) | |
| 288–303 | 908.4434 | 1814.8722 | 1814.8659 | 3 | 1 | K.YADLSDAANRNHEALR.Q (Ions score 19) | |
| 314–327 | 558.9593 | 1673.856 | 1673.8519 | 2 | 1 | R.RQIQSLTCEVDGLR.G (Ions score 25) | |
| 315–327 | 759.8845 | 1517.7544 | 1517.7508 | 2 | 0 | R.QIQSLTCEVDGLR.G (Ions score 51) | |
| 375–383 | 568.3051 | 1134.5955 | 1134.5921 | 3 | 0 | R.EYQELLNVK.M (Ions score 39) | |
| 375–383 | 590.7916 | 1179.5686 | 1179.5771 | −7 | 0 | R.EYQELLNVK.M Nitro (Y) (Ions score 7) | |
| 404–417 | 495.6216 | 1483.8429 | 1483.8399 | 2 | 0 | R.ISVPVHSFASLSLK.T (Ions score 17) | |
| 418–432 | 560.2739 | 1677.7998 | 1677.7958 | 2 | 0 | K.TTVPEVEPPQDSHSR.K (Ions score 10) | |
| 418–432 | 839.9081 | 1677.8017 | 1677.7958 | 4 | 0 | K.TTVPEVEPPQDSHSR.K (Ions score 53) | |
| 418–433 | 452.4817 | 1805.8979 | 1805.8908 | 4 | 1 | K.TTVPEVEPPQDSHSRK.M (Ions score 18) | |
| 418–433 | 602.9734 | 1805.8985 | 1805.8908 | 4 | 1 | K.TTVPEVEPPQDSHSRK.M (Ions score 16) | |
| 433–438 | 380.25 | 758.4854 | 758.4836 | 2 | 1 | R.KMVLIR.T (Ions score 37) | |
| 433–438 | 388.2478 | 774.481 | 774.4786 | 3 | 1 | R.KMVLIR.T Oxidation (M) (Ions score 28) | |
| 434–438 | 316.2024 | 630.3903 | 630.3887 | 3 | 0 | K.MVLIR.T (Ions score 24) | |
| 444–467 | 683.8236 | 2731.2653 | 2731.2573 | 3 | 2 | R.DGEVVTESQKEQHSELDKSSIHSY—(Ions score 3) |  |
| 454–467 | 553.9271 | 1658.7595 | 1658.7536 | 4 | 1 | K.EQHSELDKSSIHSY—(Ions score 27) |  |

**Table S1.** *Cont.*

| gi\|149032095 peripherin 1, isoform CRA_d [*Rattus norvegicus*] | | | | | | | | | | | | |  |
| --- | --- | --- | --- | --- | --- | --- | --- | --- | --- | --- | --- | --- | --- |
| **Start–End** | | **Observed** | | **Mr (expt)** | | **Mr (calc)** | | **ppm** | | **Miss** | | **Sequence** | |
| 26–45 | 676.9954 | | 2027.9643 | | 2027.9589 | | 3 | | 0 | | R.TFGPPPSLSPGAFSYSSSSR.F (Ions score 24) | |  |
| 26–45 | 1014.9897 | | 2027.9649 | | 2027.9589 | | 3 | | 0 | | R.TFGPPPSLSPGAFSYSSSSR.F (Ions score 66) | |  |
| 26–45 | 1014.99 | | 2027.9654 | | 2027.9589 | | 3 | | 0 | | R.TFGPPPSLSPGAFSYSSSSR.F (Ions score 81) | | |
| 51–62 | 599.7783 | | 1197.5421 | | 1197.5391 | | 3 | | 0 | | R.LLGSGSPSSSAR.L Phospho (ST) (Ions score 23) | | |
| 71–80 | 535.3108 | | 1068.607 | | 1068.604 | | 3 | | 1 | | R.AGALRLPSER.L (Ions score 21) | | |
| 71–80 | 357.2097 | | 1068.6072 | | 1068.604 | | 3 | | 1 | | R.AGALRLPSER.L (Ions score 23) | | |
| 98–110 | 801.8919 | | 1601.7693 | | 1601.7645 | | 3 | | 1 | | R.SNEKQELQELNDR.F (Ions score 75) | | |
| 98–110 | 534.9304 | | 1601.7694 | | 1601.7645 | | 3 | | 1 | | R.SNEKQELQELNDR.F (Ions score 50) | | |
| 111–117 | 434.7328 | | 867.4511 | | 867.449 | | 2 | | 0 | | R.FANFIEK.V (Ions score 37) | | |
| 111–117 | 434.733 | | 867.4515 | | 867.449 | | 3 | | 0 | | R.FANFIEK.V (Ions score 16) | | |
| 120–129 | 595.3218 | | 1188.6291 | | 1188.6251 | | 3 | | 0 | | R.FLEQQNAALR.G (Ions score 57) | | |
| 120–136 | 644.3434 | | 1930.0083 | | 1930.002 | | 3 | | 1 | | R.FLEQQNAALRGELSQAR.G (Ions score 45) | | |
| 143–152 | 630.8054 | | 1259.5963 | | 1259.5928 | | 3 | | 0 | | R.ADQLCQQELR.E (Ions score 41) | | |
| 156–163 | 493.2941 | | 984.5736 | | 984.5716 | | 2 | | 1 | | R.RELELLGR.E (Ions score 22) | | |
| 156–165 | 635.8643 | | 1269.714 | | 1269.7153 | | −1 | | 2 | | R.RELELLGRER.D (Ions score 1) | | |
| 157–163 | 415.2432 | | 828.4719 | | 828.4705 | | 2 | | 0 | | R.ELELLGR.E (Ions score 42) | | |
| 173–183 | 551.2944 | | 1100.5742 | | 1100.5713 | | 3 | | 0 | | R.DGLAEDLGALK.Q (Ions score 46) | | |
| 173–183 | 551.2952 | | 1100.5758 | | 1100.5713 | | 4 | | 0 | | R.DGLAEDLGALK.Q (Ions score 42) | | |
| 173–185 | 462.5856 | | 1384.735 | | 1384.731 | | 3 | | 1 | | R.DGLAEDLGALKQR.S (Ions score 9) | | |
| 195–212 | 770.6401 | | 2308.8984 | | 2308.9136 | | −7 | | 1 | | R.HSLCSPCLSPRDLTTIGR.G 3 Phospho (ST) (Ions score 3) | | |

**Table S2.** Identified tryptic peptides in sample 2.

| gi\|149032093 peripherin 1, isoform CRA_b [*Rattus norvegicus*] | | | | | | |
| --- | --- | --- | --- | --- | --- | --- |
| **Start–End** | **Observed** | **Mr(expt)** | **Mr(calc)** | **ppm** | **Miss** | **Sequence** |
| 2–15 | 480.8951 | 1439.6635 | 1439.6575 | 4 | 0 | M.PSSASMSHHSSGLR.S (Ions score 44) |
| 2–15 | 486.227 | 1455.6591 | 1455.6525 | 5 | 0 | M.PSSASMSHHSSGLR.S Oxidation (M) (Ions score 6) |
| 2–15 | 486.2271 | 1455.6595 | 1455.6525 | 5 | 0 | M.PSSASMSHHSSGLR.S Oxidation (M) (Ions score 9) |
| 2–15 | 486.228 | 1455.6623 | 1455.6525 | 7 | 0 | M.PSSASMSHHSSGLR.S Oxidation (M) (Ions score 4) |
| 16–25 | 381.865 | 1142.5733 | 1142.568 | 5 | 1 | R.SSISSTSYRR.T (Ions score 9) |
| 25–45 | 729.0297 | 2184.0672 | 2184.06 | 3 | 1 | R.RTFGPPPSLSPGAFSYSSSSR.F (Ions score 66) |
| 25–45 | 729.0297 | 2184.0672 | 2184.06 | 3 | 1 | R.RTFGPPPSLSPGAFSYSSSSR.F (Ions score 77) |
| 25–45 | 729.0298 | 2184.0675 | 2184.06 | 3 | 1 | R.RTFGPPPSLSPGAFSYSSSSR.F (Ions score 45) |
| 26–45 | 1014.9895 | 2027.9644 | 2027.9589 | 3 | 0 | R.TFGPPPSLSPGAFSYSSSSR.F (Ions score 75) |
| 26–45 | 1014.99 | 2027.9655 | 2027.9589 | 3 | 0 | R.TFGPPPSLSPGAFSYSSSSR.F (Ions score 76) |
| 26–45 | 1014.9904 | 2027.9662 | 2027.9589 | 4 | 0 | R.TFGPPPSLSPGAFSYSSSSR.F (Ions score 68) |
| 26–45 | 1014.9905 | 2027.9664 | 2027.9589 | 4 | 0 | R.TFGPPPSLSPGAFSYSSSSR.F (Ions score 79) |
| 26–45 | 676.9962 | 2027.9668 | 2027.9589 | 4 | 0 | R.TFGPPPSLSPGAFSYSSSSR.F (Ions score 53) |
| 26–45 | 676.9963 | 2027.9672 | 2027.9589 | 4 | 0 | R.TFGPPPSLSPGAFSYSSSSR.F (Ions score 19) |
| 26–45 | 1014.9909 | 2027.9673 | 2027.9589 | 4 | 0 | R.TFGPPPSLSPGAFSYSSSSR.F (Ions score 96) |
| 26–45 | 1014.9916 | 2027.9686 | 2027.9589 | 5 | 0 | R.TFGPPPSLSPGAFSYSSSSR.F (Ions score 87) |
| 26–45 | 1054.9736 | 2107.9327 | 2107.9252 | 4 | 0 | R.TFGPPPSLSPGAFSYSSSSR.F Phospho (ST) (Ions score 46) |
| 26–45 | 1054.974 | 2107.9334 | 2107.9252 | 4 | 0 | R.TFGPPPSLSPGAFSYSSSSR.F Phospho (ST) (Ions score 60) |
| 26–45 | 1054.9741 | 2107.9337 | 2107.9252 | 4 | 0 | R.TFGPPPSLSPGAFSYSSSSR.F Phospho (ST) (Ions score 50) |
| 46–62 | 614.9344 | 1841.7815 | 1841.771 | 6 | 1 | R.FSSSRLLGSGSPSSSAR.L 2 Phospho (ST) (Ions score 4) |
| 51–62 | 559.7954 | 1117.5763 | 1117.5727 | 3 | 0 | R.LLGSGSPSSSAR.L (Ions score 38) |
| 51–62 | 599.7789 | 1197.5432 | 1197.5391 | 3 | 0 | R.LLGSGSPSSSAR.L Phospho (ST) (Ions score 35) |
| 71–80 | 357.2098 | 1068.6077 | 1068.604 | 3 | 1 | R.AGALRLPSER.L (Ions score 35) |
| 71–80 | 535.3112 | 1068.6079 | 1068.604 | 4 | 1 | R.AGALRLPSER.L (Ions score 20) |
| 76–80 | 301.1701 | 600.3257 | 600.3231 | 4 | 0 | R.LPSER.L (Ions score 18) |
| 81–97 | 986.4808 | 1970.947 | 1970.9407 | 3 | 0 | R.LDFSMAEALNQEFLATR.S Oxidation (M) (Ions score 57) |
| 81–97 | 657.9896 | 1970.947 | 1970.9407 | 3 | 0 | R.LDFSMAEALNQEFLATR.S Oxidation (M) (Ions score 15) |
| 81–97 | 986.4811 | 1970.9477 | 1970.9407 | 4 | 0 | R.LDFSMAEALNQEFLATR.S Oxidation (M) (Ions score 42) |
| 98–110 | 801.8923 | 1601.77 | 1601.7645 | 3 | 1 | R.SNEKQELQELNDR.F (Ions score 70) |
| 98–110 | 534.9307 | 1601.7702 | 1601.7645 | 4 | 1 | R.SNEKQELQELNDR.F (Ions score 47) |
| 111–117 | 434.7324 | 867.4503 | 867.449 | 2 | 0 | R.FANFIEK.V (Ions score 38) |
| 111–117 | 434.7331 | 867.4516 | 867.449 | 3 | 0 | R.FANFIEK.V (Ions score 33) |
| 111–119 | 375.2139 | 1122.6198 | 1122.6186 | 1 | 1 | R.FANFIEKVR.F (Ions score 24) |
| 120–129 | 595.3216 | 1188.6286 | 1188.6251 | 3 | 0 | R.FLEQQNAALR.G (Ions score 35) |
| 120–129 | 595.3218 | 1188.629 | 1188.6251 | 3 | 0 | R.FLEQQNAALR.G (Ions score 46) |
| 120–129 | 595.3218 | 1188.6291 | 1188.6251 | 3 | 0 | R.FLEQQNAALR.G (Ions score 34) |
| 120–129 | 595.3218 | 1188.6291 | 1188.6251 | 3 | 0 | R.FLEQQNAALR.G (Ions score 24) |
| 120–129 | 595.322 | 1188.6294 | 1188.6251 | 4 | 0 | R.FLEQQNAALR.G (Ions score 42) |
| 120–129 | 595.322 | 1188.6294 | 1188.6251 | 4 | 0 | R.FLEQQNAALR.G (Ions score 59) |
| 120–129 | 595.3221 | 1188.6296 | 1188.6251 | 4 | 0 | R.FLEQQNAALR.G (Ions score 15) |
| 120–129 | 595.3228 | 1188.6309 | 1188.6251 | 5 | 0 | R.FLEQQNAALR.G (Ions score 19) |
| 120–136 | 644.3434 | 1930.0083 | 1930.002 | 3 | 1 | R.FLEQQNAALRGELSQAR.G (Ions score 34) |
| 120–136 | 966.0116 | 1930.0086 | 1930.002 | 3 | 1 | R.FLEQQNAALRGELSQAR.G (Ions score 11) |
| 130–136 | 380.7027 | 759.3909 | 759.3875 | 4 | 0 | R.GELSQAR.G (Ions score 31) |
| 137–152 | 633.6444 | 1897.9112 | 1897.9064 | 3 | 1 | R.GQEPARADQLCQQELR.E (Ions score 21) |
| 137–152 | 949.9633 | 1897.912 | 1897.9064 | 3 | 1 | R.GQEPARADQLCQQELR.E (Ions score 29) |
| 143–152 | 630.8058 | 1259.5971 | 1259.5928 | 3 | 0 | R.ADQLCQQELR.E (Ions score 23) |
| 143–152 | 630.8059 | 1259.5973 | 1259.5928 | 4 | 0 | R.ADQLCQQELR.E (Ions score 48) |
| 156–163 | 493.2942 | 984.5739 | 984.5716 | 2 | 1 | R.RELELLGR.E (Ions score 25) |
| 156–163 | 493.2948 | 984.575 | 984.5716 | 4 | 1 | R.RELELLGR.E (Ions score 47) |
| 156–163 | 329.199 | 984.5751 | 984.5716 | 4 | 1 | R.RELELLGR.E (Ions score 28) |
| 157–163 | 415.2435 | 828.4725 | 828.4705 | 2 | 0 | R.ELELLGR.E (Ions score 4) |
| 157–163 | 415.2436 | 828.4726 | 828.4705 | 3 | 0 | R.ELELLGR.E (Ions score 17) |
| 157–163 | 415.2437 | 828.4727 | 828.4705 | 3 | 0 | R.ELELLGR.E (Ions score 22) |
| 157–163 | 415.2437 | 828.4728 | 828.4705 | 3 | 0 | R.ELELLGR.E (Ions score 15) |
| 157–163 | 415.2439 | 828.4732 | 828.4705 | 3 | 0 | R.ELELLGR.E (Ions score 8) |
| 157–163 | 415.2441 | 828.4737 | 828.4705 | 4 | 0 | R.ELELLGR.E (Ions score 41) |
| 166–172 | 451.2484 | 900.4821 | 900.4777 | 5 | 1 | R.DRVQVER.D (Ions score 33) |
| 166–183 | 496.7683 | 1983.0439 | 1983.0385 | 3 | 2 | R.DRVQVERDGLAEDLGALK.Q (Ions score 29) |
| 166–183 | 662.022 | 1983.0443 | 1983.0385 | 3 | 2 | R.DRVQVERDGLAEDLGALK.Q (Ions score 25) |
| 168–172 | 315.6837 | 629.3527 | 629.3497 | 5 | 0 | R.VQVER.D (Ions score 20) |
| 168–183 | 856.9656 | 1711.9167 | 1711.9104 | 4 | 1 | R.VQVERDGLAEDLGALK.Q (Ions score 57) |
| 168–183 | 571.6468 | 1711.9185 | 1711.9104 | 5 | 1 | R.VQVERDGLAEDLGALK.Q (Ions score 37) |

**Table S2.** *Cont.*

| 173–183 | 551.2941 | 1100.5737 | 1100.5713 | 2 | 0 | R.DGLAEDLGALK.Q (Ions score 60) |
| --- | --- | --- | --- | --- | --- | --- |
| 173–183 | 551.2943 | 1100.5739 | 1100.5713 | 2 | 0 | R.DGLAEDLGALK.Q (Ions score 43) |
| 173–183 | 551.2944 | 1100.5743 | 1100.5713 | 3 | 0 | R.DGLAEDLGALK.Q (Ions score 44) |
| 173–183 | 551.2946 | 1100.5746 | 1100.5713 | 3 | 0 | R.DGLAEDLGALK.Q (Ions score 7) |
| 173–183 | 551.2947 | 1100.5748 | 1100.5713 | 3 | 0 | R.DGLAEDLGALK.Q (Ions score 53) |
| 173–183 | 551.2947 | 1100.5748 | 1100.5713 | 3 | 0 | R.DGLAEDLGALK.Q (Ions score 24) |
| 173–183 | 551.2947 | 1100.5748 | 1100.5713 | 3 | 0 | R.DGLAEDLGALK.Q (Ions score 16) |
| 173–183 | 551.2949 | 1100.5752 | 1100.5713 | 3 | 0 | R.DGLAEDLGALK.Q (Ions score 56) |
| 173–183 | 551.2949 | 1100.5753 | 1100.5713 | 4 | 0 | R.DGLAEDLGALK.Q (Ions score 29) |
| 173–183 | 551.295 | 1100.5754 | 1100.5713 | 4 | 0 | R.DGLAEDLGALK.Q (Ions score 73) |
| 173–183 | 551.295 | 1100.5754 | 1100.5713 | 4 | 0 | R.DGLAEDLGALK.Q (Ions score 27) |
| 173–185 | 693.3721 | 1384.7297 | 1384.731 | −1 | 1 | R.DGLAEDLGALKQR.L (Ions score 40) |
| 173–185 | 462.5857 | 1384.7353 | 1384.731 | 3 | 1 | R.DGLAEDLGALKQR.L (Ions score 16) |
| 186–191 | 388.6947 | 775.3748 | 775.3712 | 5 | 0 | R.LEEETR.K (Ions score 26) |
| 186–191 | 388.695 | 775.3755 | 775.3712 | 6 | 0 | R.LEEETR.K (Ions score 28) |
| 192–204 | 407.474 | 1625.8669 | 1625.8638 | 2 | 2 | R.KREDAEHNLVLFR.K (Ions score 8) |
| 192–204 | 407.4742 | 1625.8676 | 1625.8638 | 2 | 2 | R.KREDAEHNLVLFR.K (Ions score 24) |
| 192–204 | 542.9634 | 1625.8683 | 1625.8638 | 3 | 2 | R.KREDAEHNLVLFR.K (Ions score 13) |
| 192–204 | 542.9634 | 1625.8683 | 1625.8638 | 3 | 2 | R.KREDAEHNLVLFR.K (Ions score 5) |
| 192–204 | 407.4744 | 1625.8685 | 1625.8638 | 3 | 2 | R.KREDAEHNLVLFR.K (Ions score 25) |
| 192–204 | 407.4744 | 1625.8686 | 1625.8638 | 3 | 2 | R.KREDAEHNLVLFR.K (Ions score 13) |
| 192–204 | 542.9636 | 1625.8689 | 1625.8638 | 3 | 2 | R.KREDAEHNLVLFR.K (Ions score 30) |
| 192–204 | 407.4746 | 1625.8691 | 1625.8638 | 3 | 2 | R.KREDAEHNLVLFR.K (Ions score 23) |
| 192–204 | 407.4746 | 1625.8691 | 1625.8638 | 3 | 2 | R.KREDAEHNLVLFR.K (Ions score 22) |
| 192–204 | 407.4746 | 1625.8695 | 1625.8638 | 4 | 2 | R.KREDAEHNLVLFR.K (Ions score 9) |
| 192–204 | 407.4747 | 1625.8697 | 1625.8638 | 4 | 2 | R.KREDAEHNLVLFR.K (Ions score 48) |
| 192–204 | 542.9639 | 1625.8698 | 1625.8638 | 4 | 2 | R.KREDAEHNLVLFR.K (Ions score 35) |
| 192–204 | 407.4747 | 1625.8698 | 1625.8638 | 4 | 2 | R.KREDAEHNLVLFR.K (Ions score 27) |
| 192–204 | 407.4747 | 1625.8698 | 1625.8638 | 4 | 2 | R.KREDAEHNLVLFR.K (Ions score 32) |

**Table S2.** *Cont.*

| 192–204 | 407.4748 | 1625.8701 | 1625.8638 | 4 | 2 | R.KREDAEHNLVLFR.K (Ions score 23) |
| --- | --- | --- | --- | --- | --- | --- |
| 192–204 | 813.9423 | 1625.8701 | 1625.8638 | 4 | 2 | R.KREDAEHNLVLFR.K (Ions score 49) |
| 192–204 | 407.4749 | 1625.8705 | 1625.8638 | 4 | 2 | R.KREDAEHNLVLFR.K (Ions score 3) |
| 192–204 | 542.9643 | 1625.871 | 1625.8638 | 4 | 2 | R.KREDAEHNLVLFR.K (Ions score 14) |
| 193–204 | 500.2654 | 1497.7743 | 1497.7688 | 4 | 1 | K.REDAEHNLVLFR.K (Ions score 42) |
| 193–204 | 749.8947 | 1497.7749 | 1497.7688 | 4 | 1 | K.REDAEHNLVLFR.K (Ions score 17) |
| 193–204 | 375.451 | 1497.775 | 1497.7688 | 4 | 1 | K.REDAEHNLVLFR.K (Ions score 14) |
| 194–204 | 448.2315 | 1341.6726 | 1341.6677 | 4 | 0 | R.EDAEHNLVLFR.K (Ions score 18) |
| 194–204 | 671.8437 | 1341.6728 | 1341.6677 | 4 | 0 | R.EDAEHNLVLFR.K (Ions score 49) |
| 194–204 | 671.8439 | 1341.6732 | 1341.6677 | 4 | 0 | R.EDAEHNLVLFR.K (Ions score 45) |
| 205–219 | 587.3134 | 1758.9184 | 1758.9112 | 4 | 2 | R.KDVDDATLSRLELER.K (Ions score 12) |
| 206–214 | 496.2399 | 990.4652 | 990.4618 | 3 | 0 | K.DVDDATLSR.L (Ions score 36) |
| 206–219 | 544.6144 | 1630.8215 | 1630.8162 | 3 | 1 | K.DVDDATLSRLELER.K (Ions score 37) |
| 206–219 | 816.4183 | 1630.8221 | 1630.8162 | 4 | 1 | K.DVDDATLSRLELER.K (Ions score 26) |
| 206–219 | 816.4188 | 1630.8231 | 1630.8162 | 4 | 1 | K.DVDDATLSRLELER.K (Ions score 14) |
| 215–219 | 330.191 | 658.3674 | 658.365 | 4 | 0 | R.LELER.K (Ions score 26) |
| 220–232 | 805.9139 | 1609.8133 | 1609.8273 | −9 | 1 | R.KIESLMDEIEFLK.K Oxidation (M) (Ions score 3) |
| 220–232 | 805.9235 | 1609.8325 | 1609.8273 | 3 | 1 | R.KIESLMDEIEFLK.K Oxidation (M) (Ions score 92) |
| 220–232 | 537.6182 | 1609.8327 | 1609.8273 | 3 | 1 | R.KIESLMDEIEFLK.K Oxidation (M) (Ions score 48) |
| 220–232 | 805.9238 | 1609.833 | 1609.8273 | 4 | 1 | R.KIESLMDEIEFLK.K Oxidation (M) (Ions score 82) |
| 220–233 | 580.3163 | 1737.927 | 1737.9222 | 3 | 2 | R.KIESLMDEIEFLKK.L Oxidation (M) (Ions score 28) |
| 220–233 | 580.3163 | 1737.927 | 1737.9222 | 3 | 2 | R.KIESLMDEIEFLKK.L Oxidation (M) (Ions score 26) |
| 220–233 | 435.4893 | 1737.9281 | 1737.9222 | 3 | 2 | R.KIESLMDEIEFLKK.L Oxidation (M) (Ions score 11) |
| 220–233 | 435.4894 | 1737.9285 | 1737.9222 | 4 | 2 | R.KIESLMDEIEFLKK.L Oxidation (M) (Ions score 11) |
| 220–233 | 580.3168 | 1737.9287 | 1737.9222 | 4 | 2 | R.KIESLMDEIEFLKK.L Oxidation (M) (Ions score 41) |
| 220–233 | 869.9718 | 1737.929 | 1737.9222 | 4 | 2 | R.KIESLMDEIEFLKK.L Oxidation (M) (Ions score 57) |
| 220–233 | 580.317 | 1737.9291 | 1737.9222 | 4 | 2 | R.KIESLMDEIEFLKK.L Oxidation (M) (Ions score 53) |
| 220–233 | 580.3171 | 1737.9294 | 1737.9222 | 4 | 2 | R.KIESLMDEIEFLKK.L Oxidation (M) (Ions score 23) |
| 221–232 | 741.8753 | 1481.7361 | 1481.7323 | 3 | 0 | K.IESLMDEIEFLK.K Oxidation (M) (Ions score 78) |
| 221–232 | 741.8759 | 1481.7373 | 1481.7323 | 3 | 0 | K.IESLMDEIEFLK.K Oxidation (M) (Ions score 64) |
| 221–233 | 537.6185 | 1609.8336 | 1609.8273 | 4 | 1 | K.IESLMDEIEFLKK.L Oxidation (M) (Ions score 27) |

**Table S2.** *Cont.*

| 233–240 | 527.2902 | 1052.5659 | 1052.5614 | 4 | 1 | K.KLHEEELR.D (Ions score 14) |
| --- | --- | --- | --- | --- | --- | --- |
| 233–240 | 351.8626 | 1052.5659 | 1052.5614 | 4 | 1 | K.KLHEEELR.D (Ions score 38) |
| 273–281 | 504.2638 | 1006.513 | 1006.5083 | 5 | 0 | R.AQYENIAAK.N (Ions score 37) |
| 282–291 | 655.3021 | 1308.5897 | 1308.5986 | −7 | 0 | K.NLQEAEEWYK.S (Ions score 16) |
| 282–291 | 655.3065 | 1308.5985 | 1308.5986 | 0 | 0 | K.NLQEAEEWYK.S (Ions score 18) |
| 282–291 | 655.3073 | 1308.6001 | 1308.5986 | 1 | 0 | K.NLQEAEEWYK.S (Ions score 18) |
| 282–291 | 655.3075 | 1308.6004 | 1308.5986 | 1 | 0 | K.NLQEAEEWYK.S (Ions score 4) |
| 282–291 | 655.3076 | 1308.6007 | 1308.5986 | 2 | 0 | K.NLQEAEEWYK.S (Ions score 31) |
| 282–291 | 655.3078 | 1308.601 | 1308.5986 | 2 | 0 | K.NLQEAEEWYK.S (Ions score 7) |
| 282–291 | 655.3079 | 1308.6013 | 1308.5986 | 2 | 0 | K.NLQEAEEWYK.S (Ions score 11) |
| 282–291 | 655.3079 | 1308.6013 | 1308.5986 | 2 | 0 | K.NLQEAEEWYK.S (Ions score 21) |
| 282–291 | 655.308 | 1308.6014 | 1308.5986 | 2 | 0 | K.NLQEAEEWYK.S (Ions score 15) |
| 282–291 | 655.3081 | 1308.6017 | 1308.5986 | 2 | 0 | K.NLQEAEEWYK.S (Ions score 19) |
| 282–291 | 655.3083 | 1308.602 | 1308.5986 | 3 | 0 | K.NLQEAEEWYK.S (Ions score 24) |
| 282–291 | 655.3084 | 1308.6021 | 1308.5986 | 3 | 0 | K.NLQEAEEWYK.S (Ions score 15) |
| 282–291 | 655.3084 | 1308.6023 | 1308.5986 | 3 | 0 | K.NLQEAEEWYK.S (Ions score 15) |
| 282–291 | 655.3085 | 1308.6024 | 1308.5986 | 3 | 0 | K.NLQEAEEWYK.S (Ions score 23) |
| 282–291 | 655.3085 | 1308.6025 | 1308.5986 | 3 | 0 | K.NLQEAEEWYK.S (Ions score 42) |
| 282–291 | 655.3087 | 1308.6029 | 1308.5986 | 3 | 0 | K.NLQEAEEWYK.S (Ions score 49) |
| 282–291 | 655.3087 | 1308.6029 | 1308.5986 | 3 | 0 | K.NLQEAEEWYK.S (Ions score 25) |
| 282–291 | 655.3088 | 1308.603 | 1308.5986 | 3 | 0 | K.NLQEAEEWYK.S (Ions score 14) |
| 282–291 | 655.3088 | 1308.6031 | 1308.5986 | 3 | 0 | K.NLQEAEEWYK.S (Ions score 16) |
| 282–291 | 655.3088 | 1308.6031 | 1308.5986 | 3 | 0 | K.NLQEAEEWYK.S (Ions score 24) |
| 282–291 | 655.3089 | 1308.6032 | 1308.5986 | 4 | 0 | K.NLQEAEEWYK.S (Ions score 11) |
| 282–291 | 655.309 | 1308.6034 | 1308.5986 | 4 | 0 | K.NLQEAEEWYK.S (Ions score 27) |
| 282–291 | 655.3091 | 1308.6037 | 1308.5986 | 4 | 0 | K.NLQEAEEWYK.S (Ions score 22) |
| 282–291 | 655.3094 | 1308.6042 | 1308.5986 | 4 | 0 | K.NLQEAEEWYK.S (Ions score 9) |
| 282–291 | 655.3094 | 1308.6043 | 1308.5986 | 4 | 0 | K.NLQEAEEWYK.S (Ions score 11) |
| 282–291 | 655.3099 | 1308.6052 | 1308.5986 | 5 | 0 | K.NLQEAEEWYK.S (Ions score 2) |
| 282–291 | 655.3101 | 1308.6056 | 1308.5986 | 5 | 0 | K.NLQEAEEWYK.S (Ions score 9) |
| 282–291 | 655.3111 | 1308.6076 | 1308.5986 | 7 | 0 | K.NLQEAEEWYK.S (Ions score 5) |

**Table S2.** *Cont.*

| 292–303 | 655.8225 | 1309.6303 | 1309.6262 | 3 | 1 | K.SKYADLSDAANR.N (Ions score 66) |
| --- | --- | --- | --- | --- | --- | --- |
| 292–303 | 437.5509 | 1309.6308 | 1309.6262 | 3 | 1 | K.SKYADLSDAANR.N (Ions score 45) |
| 292–309 | 508.507 | 2029.9991 | 2029.9929 | 3 | 2 | K.SKYADLSDAANRNHEALR.Q (Ions score 29) |
| 294–303 | 548.2583 | 1094.502 | 1094.4992 | 3 | 0 | K.YADLSDAANR.N (Ions score 32) |
| 294–303 | 548.2585 | 1094.5025 | 1094.4992 | 3 | 0 | K.YADLSDAANR.N (Ions score 11) |
| 294–303 | 548.2587 | 1094.5028 | 1094.4992 | 3 | 0 | K.YADLSDAANR.N (Ions score 52) |
| 294–303 | 548.259 | 1094.5034 | 1094.4992 | 4 | 0 | K.YADLSDAANR.N (Ions score 54) |
| 294–303 | 548.2592 | 1094.5038 | 1094.4992 | 4 | 0 | K.YADLSDAANR.N (Ions score 50) |
| 294–303 | 548.2609 | 1094.5072 | 1094.4992 | 7 | 0 | K.YADLSDAANR.N (Ions score 5) |
| 294–309 | 605.9645 | 1814.8718 | 1814.8659 | 3 | 1 | K.YADLSDAANRNHEALR.Q (Ions score 36) |
| 294–309 | 454.7253 | 1814.8723 | 1814.8659 | 3 | 1 | K.YADLSDAANRNHEALR.Q (Ions score 27) |
| 294–309 | 908.4435 | 1814.8725 | 1814.8659 | 4 | 1 | K.YADLSDAANRNHEALR.Q (Ions score 22) |
| 320–333 | 558.9592 | 1673.8557 | 1673.8519 | 2 | 1 | R.RQIQSLTCEVDGLR.G (Ions score 21) |
| 320–333 | 558.9596 | 1673.8569 | 1673.8519 | 3 | 1 | R.RQIQSLTCEVDGLR.G (Ions score 32) |
| 320–333 | 558.96 | 1673.8581 | 1673.8519 | 4 | 1 | R.RQIQSLTCEVDGLR.G (Ions score 43) |
| 320–333 | 837.9364 | 1673.8582 | 1673.8519 | 4 | 1 | R.RQIQSLTCEVDGLR.G (Ions score 48) |
| 321–333 | 759.8834 | 1517.7522 | 1517.7508 | 1 | 0 | R.QIQSLTCEVDGLR.G (Ions score 28) |
| 321–333 | 759.884 | 1517.7535 | 1517.7508 | 2 | 0 | R.QIQSLTCEVDGLR.G (Ions score 11) |
| 321–333 | 759.8851 | 1517.7557 | 1517.7508 | 3 | 0 | R.QIQSLTCEVDGLR.G (Ions score 59) |
| 334–341 | 437.2449 | 872.4752 | 872.4716 | 4 | 0 | R.GTNEALLR.Q (Ions score 56) |
| 364–369 | 394.7125 | 787.4104 | 787.4075 | 4 | 0 | R.LEEELR.Q (Ions score 30) |
| 370–377 | 335.5125 | 1003.5156 | 1003.512 | 4 | 1 | R.QLKEEMAR.H (Ions score 6) |
| 378–389 | 514.6204 | 1540.8394 | 1540.8361 | 2 | 1 | R.HLREYQELLNVK.M (Ions score 13) |
| 381–389 | 568.3044 | 1134.5942 | 1134.5921 | 2 | 0 | R.EYQELLNVK.M (Ions score 39) |
| 381–389 | 568.3047 | 1134.5948 | 1134.5921 | 2 | 0 | R.EYQELLNVK.M (Ions score 40) |
| 381–389 | 568.3051 | 1134.5957 | 1134.5921 | 3 | 0 | R.EYQELLNVK.M (Ions score 49) |
| 381–389 | 568.3067 | 1134.5988 | 1134.5921 | 6 | 0 | R.EYQELLNVK.M (Ions score 14) |
| 381–389 | 590.7911 | 1179.5676 | 1179.5771 | −8 | 0 | R.EYQELLNVK.M Nitro (Y) (Ions score 2) |

**Table S2.** *Cont.*

| 390–400 | 656.3363 | 1310.658 | 1310.654 | 3 | 0 | K.MALDIEIATYR.K Oxidation (M) (Ions score 62) |
| --- | --- | --- | --- | --- | --- | --- |
| 390–401 | 720.3832 | 1438.7518 | 1438.749 | 2 | 1 | K.MALDIEIATYRK.L Oxidation (M) (Ions score 49) |
| 390–401 | 720.3832 | 1438.7519 | 1438.749 | 2 | 1 | K.MALDIEIATYRK.L Oxidation (M) (Ions score 64) |
| 390–401 | 480.5919 | 1438.7539 | 1438.749 | 3 | 1 | K.MALDIEIATYRK.L Oxidation (M) (Ions score 37) |
| 401–409 | 530.7875 | 1059.5605 | 1059.556 | 4 | 1 | R.KLLEGEESR.I (Ions score 48) |
| 410–423 | 742.929 | 1483.8435 | 1483.8399 | 2 | 0 | R.ISVPVHSFASLSLK.T (Ions score 58) |
| 410–423 | 495.6218 | 1483.8437 | 1483.8399 | 3 | 0 | R.ISVPVHSFASLSLK.T (Ions score 22) |
| 424–438 | 839.9078 | 1677.801 | 1677.7958 | 3 | 0 | K.TTVPEVEPPQDSHSR.K (Ions score 25) |
| 424–438 | 839.908 | 1677.8015 | 1677.7958 | 3 | 0 | K.TTVPEVEPPQDSHSR.K (Ions score 37) |
| 424–438 | 560.2745 | 1677.8018 | 1677.7958 | 4 | 0 | K.TTVPEVEPPQDSHSR.K (Ions score 13) |
| 424–438 | 560.2747 | 1677.8023 | 1677.7958 | 4 | 0 | K.TTVPEVEPPQDSHSR.K (Ions score 36) |
| 424–438 | 560.2748 | 1677.8027 | 1677.7958 | 4 | 0 | K.TTVPEVEPPQDSHSR.K (Ions score 29) |
| 424–438 | 560.275 | 1677.8031 | 1677.7958 | 4 | 0 | K.TTVPEVEPPQDSHSR.K (Ions score 8) |
| 424–438 | 839.9088 | 1677.8031 | 1677.7958 | 4 | 0 | K.TTVPEVEPPQDSHSR.K (Ions score 38) |
| 424–439 | 903.956 | 1805.8974 | 1805.8908 | 4 | 1 | K.TTVPEVEPPQDSHSRK.M (Ions score 38) |
| 424–439 | 452.4818 | 1805.898 | 1805.8908 | 4 | 1 | K.TTVPEVEPPQDSHSRK.M (Ions score 17) |
| 439–444 | 380.2504 | 758.4863 | 758.4836 | 4 | 1 | R.KMVLIR.T (Ions score 36) |
| 439–444 | 388.2481 | 774.4817 | 774.4786 | 4 | 1 | R.KMVLIR.T Oxidation (M) (Ions score 24) |
| 440–444 | 316.2025 | 630.3905 | 630.3887 | 3 | 0 | K.MVLIR.T (Ions score 20) |
| 445–449 | 310.1755 | 618.3365 | 618.3337 | 5 | 0 | R.TIETR.D (Ions score 17) |
| 454–468 | 878.9434 | 1755.8722 | 1755.8639 | 5 | 1 | K.VVTESQKEQHSELDK.S (Ions score 56) |
| 454–468 | 439.9753 | 1755.8723 | 1755.8639 | 5 | 1 | K.VVTESQKEQHSELDK.S (Ions score 3) |
| 454–468 | 586.2981 | 1755.8725 | 1755.8639 | 5 | 1 | K.VVTESQKEQHSELDK.S (Ions score 36) |
| 454–474 | 1216.0941 | 2430.1737 | 2430.1663 | 3 | 2 | K.VVTESQKEQHSELDKSSIHSY—(Ions score 41) |
| 454–474 | 811.0652 | 2430.1737 | 2430.1663 | 3 | 2 | K.VVTESQKEQHSELDKSSIHSY—(Ions score 26) |
| 454–474 | 608.5515 | 2430.1767 | 2430.1663 | 4 | 2 | K.VVTESQKEQHSELDKSSIHSY—(Ions score 1) |
| 454–474 | 837.7215 | 2510.1427 | 2510.1326 | 4 | 2 | K.VVTESQKEQHSELDKSSIHSY—Phospho (ST) (Ions score 6) |
| 461–474 | 830.3872 | 1658.7599 | 1658.7536 | 4 | 1 | K.EQHSELDKSSIHSY—(Ions score 39) |
| 461–474 | 553.9272 | 1658.7599 | 1658.7536 | 4 | 1 | K.EQHSELDKSSIHSY—(Ions score 39) |
| 461–474 | 415.6973 | 1658.7602 | 1658.7536 | 4 | 1 | K.EQHSELDKSSIHSY—(Ions score 15) |

**Table S2.** *Cont.*

| gi\|166063971 peripherin [*Rattus norvegicus*] | | | | | | |
| --- | --- | --- | --- | --- | --- | --- |
| **Start–End** | **Observed** | **Mr (expt)** | **Mr (calc)** | **ppm** | **Miss** | **Sequence** |
| 2–15 | 480.8951 | 1439.6635 | 1439.6575 | 4 | 0 | M.PSSASMSHHSSGLR.S (Ions score 44) |
| 2–15 | 486.227 | 1455.6591 | 1455.6525 | 5 | 0 | M.PSSASMSHHSSGLR.S Oxidation (M) (Ions score 6) |
| 2–15 | 486.2271 | 1455.6595 | 1455.6525 | 5 | 0 | M.PSSASMSHHSSGLR.S Oxidation (M) (Ions score 9) |
| 2–15 | 486.228 | 1455.6623 | 1455.6525 | 7 | 0 | M.PSSASMSHHSSGLR.S Oxidation (M) (Ions score 4) |
| 16–25 | 381.865 | 1142.5733 | 1142.568 | 5 | 1 | R.SSISSTSYRR.T (Ions score 9) |
| 25–45 | 729.0297 | 2184.0672 | 2184.06 | 3 | 1 | R.RTFGPPPSLSPGAFSYSSSSR.F (Ions score 66) |
| 25–45 | 729.0297 | 2184.0672 | 2184.06 | 3 | 1 | R.RTFGPPPSLSPGAFSYSSSSR.F (Ions score 77) |
| 25–45 | 729.0298 | 2184.0675 | 2184.06 | 3 | 1 | R.RTFGPPPSLSPGAFSYSSSSR.F (Ions score 45) |
| 26–45 | 1014.9895 | 2027.9644 | 2027.9589 | 3 | 0 | R.TFGPPPSLSPGAFSYSSSSR.F (Ions score 75) |
| 26–45 | 1014.99 | 2027.9655 | 2027.9589 | 3 | 0 | R.TFGPPPSLSPGAFSYSSSSR.F (Ions score 76) |
| 26–45 | 1014.9904 | 2027.9662 | 2027.9589 | 4 | 0 | R.TFGPPPSLSPGAFSYSSSSR.F (Ions score 68) |
| 26–45 | 1014.9905 | 2027.9664 | 2027.9589 | 4 | 0 | R.TFGPPPSLSPGAFSYSSSSR.F (Ions score 79) |
| 26–45 | 676.9962 | 2027.9668 | 2027.9589 | 4 | 0 | R.TFGPPPSLSPGAFSYSSSSR.F (Ions score 53) |
| 26–45 | 676.9963 | 2027.9672 | 2027.9589 | 4 | 0 | R.TFGPPPSLSPGAFSYSSSSR.F (Ions score 19) |
| 26–45 | 1014.9909 | 2027.9673 | 2027.9589 | 4 | 0 | R.TFGPPPSLSPGAFSYSSSSR.F (Ions score 96) |
| 26–45 | 1014.9916 | 2027.9686 | 2027.9589 | 5 | 0 | R.TFGPPPSLSPGAFSYSSSSR.F (Ions score 87) |
| 26–45 | 1054.9736 | 2107.9327 | 2107.9252 | 4 | 0 | R.TFGPPPSLSPGAFSYSSSSR.F Phospho (ST) (Ions score 46) |
| 26–45 | 1054.974 | 2107.9334 | 2107.9252 | 4 | 0 | R.TFGPPPSLSPGAFSYSSSSR.F Phospho (ST) (Ions score 60) |
| 26–45 | 1054.9741 | 2107.9337 | 2107.9252 | 4 | 0 | R.TFGPPPSLSPGAFSYSSSSR.F Phospho (ST) (Ions score 50) |
| 46–62 | 614.9344 | 1841.7815 | 1841.771 | 6 | 1 | R.FSSSRLLGSGSPSSSAR.L 2 Phospho (ST) (Ions score 4) |
| 51–62 | 559.7954 | 1117.5763 | 1117.5727 | 3 | 0 | R.LLGSGSPSSSAR.L (Ions score 38) |
| 51–62 | 599.7789 | 1197.5432 | 1197.5391 | 3 | 0 | R.LLGSGSPSSSAR.L Phospho (ST) (Ions score 35) |
| 71–80 | 357.2098 | 1068.6077 | 1068.604 | 3 | 1 | R.AGALRLPSER.L (Ions score 35) |
| 71–80 | 535.3112 | 1068.6079 | 1068.604 | 4 | 1 | R.AGALRLPSER.L (Ions score 20) |
| 76–80 | 301.1701 | 600.3257 | 600.3231 | 4 | 0 | R.LPSER.L (Ions score 18) |
| 81–97 | 986.4808 | 1970.947 | 1970.9407 | 3 | 0 | R.LDFSMAEALNQEFLATR.S Oxidation (M) (Ions score 57) |
| 81–97 | 657.9896 | 1970.947 | 1970.9407 | 3 | 0 | R.LDFSMAEALNQEFLATR.S Oxidation (M) (Ions score 15) |
| 81–97 | 986.4811 | 1970.9477 | 1970.9407 | 4 | 0 | R.LDFSMAEALNQEFLATR.S Oxidation (M) (Ions score 42) |

**Table S2.** *Cont.*

| 98–110 | 801.8923 | 1601.77 | 1601.7645 | 3 | 1 | R.SNEKQELQELNDR.F (Ions score 70) |
| --- | --- | --- | --- | --- | --- | --- |
| 98–110 | 534.9307 | 1601.7702 | 1601.7645 | 4 | 1 | R.SNEKQELQELNDR.F (Ions score 47) |
| 111–117 | 434.7324 | 867.4503 | 867.449 | 2 | 0 | R.FANFIEK.V (Ions score 38) |
| 111–117 | 434.7331 | 867.4516 | 867.449 | 3 | 0 | R.FANFIEK.V (Ions score 33) |
| 111–119 | 375.2139 | 1122.6198 | 1122.6186 | 1 | 1 | R.FANFIEKVR.F (Ions score 24) |
| 120–129 | 595.3216 | 1188.6286 | 1188.6251 | 3 | 0 | R.FLEQQNAALR.G (Ions score 35) |
| 120–129 | 595.3218 | 1188.629 | 1188.6251 | 3 | 0 | R.FLEQQNAALR.G (Ions score 46) |
| 120–129 | 595.3218 | 1188.6291 | 1188.6251 | 3 | 0 | R.FLEQQNAALR.G (Ions score 34) |
| 120–129 | 595.3218 | 1188.6291 | 1188.6251 | 3 | 0 | R.FLEQQNAALR.G (Ions score 24) |
| 120–129 | 595.3220 | 1188.6294 | 1188.6251 | 4 | 0 | R.FLEQQNAALR.G (Ions score 42) |
| 120–129 | 595.3220 | 1188.6294 | 1188.6251 | 4 | 0 | R.FLEQQNAALR.G (Ions score 59) |
| 120–129 | 595.3221 | 1188.6296 | 1188.6251 | 4 | 0 | R.FLEQQNAALR.G (Ions score 15) |
| 120–129 | 595.3228 | 1188.6309 | 1188.6251 | 5 | 0 | R.FLEQQNAALR.G (Ions score 19) |
| 120–136 | 644.3434 | 1930.0083 | 1930.002 | 3 | 1 | R.FLEQQNAALRGELSQAR.G (Ions score 34) |
| 120–136 | 966.0116 | 1930.0086 | 1930.002 | 3 | 1 | R.FLEQQNAALRGELSQAR.G (Ions score 11) |
| 130–136 | 380.7027 | 759.3909 | 759.3875 | 4 | 0 | R.GELSQAR.G (Ions score 31) |
| 137–152 | 633.6444 | 1897.9112 | 1897.9064 | 3 | 1 | R.GQEPARADQLCQQELR.E (Ions score 21) |
| 137–152 | 949.9633 | 1897.912 | 1897.9064 | 3 | 1 | R.GQEPARADQLCQQELR.E (Ions score 29) |
| 143–152 | 630.8058 | 1259.5971 | 1259.5928 | 3 | 0 | R.ADQLCQQELR.E (Ions score 23) |
| 143–152 | 630.8059 | 1259.5973 | 1259.5928 | 4 | 0 | R.ADQLCQQELR.E (Ions score 48) |
| 156–163 | 493.2942 | 984.5739 | 984.5716 | 2 | 1 | R.RELELLGR.E (Ions score 25) |
| 156–163 | 493.2948 | 984.575 | 984.5716 | 4 | 1 | R.RELELLGR.E (Ions score 47) |
| 156–163 | 329.1990 | 984.5751 | 984.5716 | 4 | 1 | R.RELELLGR.E (Ions score 28) |
| 157–163 | 415.2435 | 828.4725 | 828.4705 | 2 | 0 | R.ELELLGR.E (Ions score 4) |
| 157–163 | 415.2436 | 828.4726 | 828.4705 | 3 | 0 | R.ELELLGR.E (Ions score 17) |
| 157–163 | 415.2437 | 828.4727 | 828.4705 | 3 | 0 | R.ELELLGR.E (Ions score 22) |
| 157–163 | 415.2437 | 828.4728 | 828.4705 | 3 | 0 | R.ELELLGR.E (Ions score 15) |
| 157–163 | 415.2439 | 828.4732 | 828.4705 | 3 | 0 | R.ELELLGR.E (Ions score 8) |
| 157–163 | 415.2441 | 828.4737 | 828.4705 | 4 | 0 | R.ELELLGR.E (Ions score 41) |
| 166–172 | 451.2484 | 900.4821 | 900.4777 | 5 | 1 | R.DRVQVER.D (Ions score 33) |

**Table S2.** *Cont.*

| 166–183 | 496.7683 | 1983.0439 | 1983.0385 | 3 | 2 | R.DRVQVERDGLAEDLGALK.Q (Ions score 29) |
| --- | --- | --- | --- | --- | --- | --- |
| 166–183 | 662.0220 | 1983.0443 | 1983.0385 | 3 | 2 | R.DRVQVERDGLAEDLGALK.Q (Ions score 25) |
| 168–172 | 315.6837 | 629.3527 | 629.3497 | 5 | 0 | R.VQVER.D (Ions score 20) |
| 168–183 | 856.9656 | 1711.9167 | 1711.9104 | 4 | 1 | R.VQVERDGLAEDLGALK.Q (Ions score 57) |
| 168–183 | 571.6468 | 1711.9185 | 1711.9104 | 5 | 1 | R.VQVERDGLAEDLGALK.Q (Ions score 37) |
| 173–183 | 551.2941 | 1100.5737 | 1100.5713 | 2 | 0 | R.DGLAEDLGALK.Q (Ions score 60) |
| 173–183 | 551.2943 | 1100.5739 | 1100.5713 | 2 | 0 | R.DGLAEDLGALK.Q (Ions score 43) |
| 173–183 | 551.2944 | 1100.5743 | 1100.5713 | 3 | 0 | R.DGLAEDLGALK.Q (Ions score 44) |
| 173–183 | 551.2946 | 1100.5746 | 1100.5713 | 3 | 0 | R.DGLAEDLGALK.Q (Ions score 7) |
| 173–183 | 551.2947 | 1100.5748 | 1100.5713 | 3 | 0 | R.DGLAEDLGALK.Q (Ions score 53) |
| 173–183 | 551.2947 | 1100.5748 | 1100.5713 | 3 | 0 | R.DGLAEDLGALK.Q (Ions score 24) |
| 173–183 | 551.2947 | 1100.5748 | 1100.5713 | 3 | 0 | R.DGLAEDLGALK.Q (Ions score 16) |
| 173–183 | 551.2949 | 1100.5752 | 1100.5713 | 3 | 0 | R.DGLAEDLGALK.Q (Ions score 56) |
| 173–183 | 551.2949 | 1100.5753 | 1100.5713 | 4 | 0 | R.DGLAEDLGALK.Q (Ions score 29) |
| 173–183 | 551.2950 | 1100.5754 | 1100.5713 | 4 | 0 | R.DGLAEDLGALK.Q (Ions score 73) |
| 173–183 | 551.2950 | 1100.5754 | 1100.5713 | 4 | 0 | R.DGLAEDLGALK.Q (Ions score 27) |
| 173–185 | 693.3721 | 1384.7297 | 1384.731 | −1 | 1 | R.DGLAEDLGALKQR.L (Ions score 40) |
| 173–185 | 462.5857 | 1384.7353 | 1384.731 | 3 | 1 | R.DGLAEDLGALKQR.L (Ions score 16) |
| 186–191 | 388.6947 | 775.3748 | 775.3712 | 5 | 0 | R.LEEETR.K (Ions score 26) |
| 186–191 | 388.6950 | 775.3755 | 775.3712 | 6 | 0 | R.LEEETR.K (Ions score 28) |
| 192–204 | 407.4740 | 1625.8669 | 1625.8638 | 2 | 2 | R.KREDAEHNLVLFR.K (Ions score 8) |
| 192–204 | 407.4742 | 1625.8676 | 1625.8638 | 2 | 2 | R.KREDAEHNLVLFR.K (Ions score 24) |
| 192–204 | 542.9634 | 1625.8683 | 1625.8638 | 3 | 2 | R.KREDAEHNLVLFR.K (Ions score 13) |
| 192–204 | 542.9634 | 1625.8683 | 1625.8638 | 3 | 2 | R.KREDAEHNLVLFR.K (Ions score 5) |
| 192–204 | 407.4744 | 1625.8685 | 1625.8638 | 3 | 2 | R.KREDAEHNLVLFR.K (Ions score 25) |
| 192–204 | 407.4744 | 1625.8686 | 1625.8638 | 3 | 2 | R.KREDAEHNLVLFR.K (Ions score 13) |
| 192–204 | 542.9636 | 1625.8689 | 1625.8638 | 3 | 2 | R.KREDAEHNLVLFR.K (Ions score 30) |
| 192–204 | 407.4746 | 1625.8691 | 1625.8638 | 3 | 2 | R.KREDAEHNLVLFR.K (Ions score 23) |
| 192–204 | 407.4746 | 1625.8691 | 1625.8638 | 3 | 2 | R.KREDAEHNLVLFR.K (Ions score 22) |

**Table S2.** *Cont.*

| 192–204 | 407.4746 | 1625.8695 | | 1625.8638 | | 4 | 2 | R.KREDAEHNLVLFR.K (Ions score 9) |
| --- | --- | --- | --- | --- | --- | --- | --- | --- |
| 192–204 | 407.4747 | 1625.8697 | | 1625.8638 | | 4 | 2 | R.KREDAEHNLVLFR.K (Ions score 48) |
| 192–204 | 542.9639 | 1625.8698 | | 1625.8638 | | 4 | 2 | R.KREDAEHNLVLFR.K (Ions score 35) |
| 192–204 | 407.4747 | 1625.8698 | | 1625.8638 | | 4 | 2 | R.KREDAEHNLVLFR.K (Ions score 27) |
| 192–204 | 407.4747 | 1625.8698 | | 1625.8638 | | 4 | 2 | R.KREDAEHNLVLFR.K (Ions score 32) |
| 192–204 | 407.4748 | 1625.8701 | | 1625.8638 | | 4 | 2 | R.KREDAEHNLVLFR.K (Ions score 23) |
| 192–204 | 813.9423 | 1625.8701 | | 1625.8638 | | 4 | 2 | R.KREDAEHNLVLFR.K (Ions score 49) |
| 192–204 | 407.4749 | 1625.8705 | | 1625.8638 | | 4 | 2 | R.KREDAEHNLVLFR.K (Ions score 3) |
| 192–204 | 542.9643 | 1625.871 | | 1625.8638 | | 4 | 2 | R.KREDAEHNLVLFR.K (Ions score 14) |
| 193–204 | 500.2654 | 1497.7743 | | 1497.7688 | | 4 | 1 | K.REDAEHNLVLFR.K (Ions score 42) |
| 193–204 | 749.8947 | 1497.7749 | | 1497.7688 | | 4 | 1 | K.REDAEHNLVLFR.K (Ions score 17) |
| 193–204 | 375.4510 | 1497.775 | | 1497.7688 | | 4 | 1 | K.REDAEHNLVLFR.K (Ions score 14) |
| 194–204 | 448.2315 | 1341.6726 | | 1341.6677 | | 4 | 0 | R.EDAEHNLVLFR.K (Ions score 18) |
| 194–204 | 671.8437 | 1341.6728 | | 1341.6677 | | 4 | 0 | R.EDAEHNLVLFR.K (Ions score 49) |
| 194–204 | 671.8439 | 1341.6732 | | 1341.6677 | | 4 | 0 | R.EDAEHNLVLFR.K (Ions score 45) |
| 205–219 | 587.3134 | 1758.9184 | | 1758.9112 | | 4 | 2 | R.KDVDDATLSRLELER.K (Ions score 12) |
| 206–214 | 496.2399 | 990.4652 | | 990.4618 | | 3 | 0 | K.DVDDATLSR.L (Ions score 36) |
| 206–219 | 544.6144 | 1630.8215 | | 1630.8162 | | 3 | 1 | K.DVDDATLSRLELER.K (Ions score 37) |
| 206–219 | 816.4183 | 1630.8221 | | 1630.8162 | | 4 | 1 | K.DVDDATLSRLELER.K (Ions score 26) |
| 206–219 | 816.4188 | | 1630.8231 | | 1630.8162 | 4 | 1 | K.DVDDATLSRLELER.K (Ions score 14) |
| 215–219 | 330.1910 | | 658.3674 | | 658.365 | 4 | 0 | R.LELER.K (Ions score 26) |
| 220–232 | 805.9139 | | 1609.8133 | | 1609.8273 | −9 | 1 | R.KIESLMDEIEFLK.K Oxidation (M) (Ions score 3) |
| 220–232 | 805.9235 | | 1609.8325 | | 1609.8273 | 3 | 1 | R.KIESLMDEIEFLK.K Oxidation (M) (Ions score 92) |
| 220–232 | 537.6182 | | 1609.8327 | | 1609.8273 | 3 | 1 | R.KIESLMDEIEFLK.K Oxidation (M) (Ions score 48) |
| 220–232 | 805.9238 | | 1609.833 | | 1609.8273 | 4 | 1 | R.KIESLMDEIEFLK.K Oxidation (M) (Ions score 82) |
| 220–233 | 580.3163 | | 1737.927 | | 1737.9222 | 3 | 2 | R.KIESLMDEIEFLKK.L Oxidation (M) (Ions score 28) |
| 220–233 | 580.3163 | | 1737.927 | | 1737.9222 | 3 | 2 | R.KIESLMDEIEFLKK.L Oxidation (M) (Ions score 26) |
| 220–233 | 435.4893 | | 1737.9281 | | 1737.9222 | 3 | 2 | R.KIESLMDEIEFLKK.L Oxidation (M) (Ions score 11) |

**Table S2.** *Cont.*

| 220–233 | 435.4894 | 1737.9285 | 1737.9222 | 4 | 2 | R.KIESLMDEIEFLKK.L Oxidation (M) (Ions score 11) |
| --- | --- | --- | --- | --- | --- | --- |
| 220–233 | 580.3168 | 1737.9287 | 1737.9222 | 4 | 2 | R.KIESLMDEIEFLKK.L Oxidation (M) (Ions score 41) |
| 220–233 | 869.9718 | 1737.929 | 1737.9222 | 4 | 2 | R.KIESLMDEIEFLKK.L Oxidation (M) (Ions score 57) |
| 220–233 | 580.3170 | 1737.9291 | 1737.9222 | 4 | 2 | R.KIESLMDEIEFLKK.L Oxidation (M) (Ions score 53) |
| 220–233 | 580.3171 | 1737.9294 | 1737.9222 | 4 | 2 | R.KIESLMDEIEFLKK.L Oxidation (M) (Ions score 23) |
| 221–232 | 741.8753 | 1481.7361 | 1481.7323 | 3 | 0 | K.IESLMDEIEFLK.K Oxidation (M) (Ions score 78) |
| 221–232 | 741.8759 | 1481.7373 | 1481.7323 | 3 | 0 | K.IESLMDEIEFLK.K Oxidation (M) (Ions score 64) |
| 221–233 | 537.6185 | 1609.8336 | 1609.8273 | 4 | 1 | K.IESLMDEIEFLKK.L Oxidation (M) (Ions score 27) |
| 233–240 | 527.2902 | 1052.5659 | 1052.5614 | 4 | 1 | K.KLHEEELR.D (Ions score 14) |
| 233–240 | 351.8626 | 1052.5659 | 1052.5614 | 4 | 1 | K.KLHEEELR.D (Ions score 38) |
| 273–281 | 504.2638 | 1006.513 | 1006.5083 | 5 | 0 | R.AQYENIAAK.N (Ions score 37) |
| 282–291 | 655.3021 | 1308.5897 | 1308.5986 | −7 | 0 | K.NLQEAEEWYK.S (Ions score 16) |
| 282–291 | 655.3065 | 1308.5985 | 1308.5986 | 0 | 0 | K.NLQEAEEWYK.S (Ions score 18) |
| 282–291 | 655.3073 | 1308.6001 | 1308.5986 | 1 | 0 | K.NLQEAEEWYK.S (Ions score 18) |
| 282–291 | 655.3075 | 1308.6004 | 1308.5986 | 1 | 0 | K.NLQEAEEWYK.S (Ions score 4) |
| 282–291 | 655.3076 | 1308.6007 | 1308.5986 | 2 | 0 | K.NLQEAEEWYK.S (Ions score 31) |
| 282–291 | 655.3078 | 1308.601 | 1308.5986 | 2 | 0 | K.NLQEAEEWYK.S (Ions score 7) |
| 282–291 | 655.3079 | 1308.6013 | 1308.5986 | 2 | 0 | K.NLQEAEEWYK.S (Ions score 11) |
| 282–291 | 655.3079 | 1308.6013 | 1308.5986 | 2 | 0 | K.NLQEAEEWYK.S (Ions score 21) |
| 282–291 | 655.3080 | 1308.6014 | 1308.5986 | 2 | 0 | K.NLQEAEEWYK.S (Ions score 15) |
| 282–291 | 655.3081 | 1308.6017 | 1308.5986 | 2 | 0 | K.NLQEAEEWYK.S (Ions score 19) |
| 282–291 | 655.3083 | 1308.602 | 1308.5986 | 3 | 0 | K.NLQEAEEWYK.S (Ions score 24) |
| 282–291 | 655.3084 | 1308.6021 | 1308.5986 | 3 | 0 | K.NLQEAEEWYK.S (Ions score 15) |
| 282–291 | 655.3084 | 1308.6023 | 1308.5986 | 3 | 0 | K.NLQEAEEWYK.S (Ions score 15) |
| 282–291 | 655.3085 | 1308.6024 | 1308.5986 | 3 | 0 | K.NLQEAEEWYK.S (Ions score 23) |
| 282–291 | 655.3085 | 1308.6025 | 1308.5986 | 3 | 0 | K.NLQEAEEWYK.S (Ions score 42) |
| 282–291 | 655.3087 | 1308.6029 | 1308.5986 | 3 | 0 | K.NLQEAEEWYK.S (Ions score 49) |
| 282–291 | 655.3087 | 1308.6029 | 1308.5986 | 3 | 0 | K.NLQEAEEWYK.S (Ions score 25) |
| 282–291 | 655.3088 | 1308.603 | 1308.5986 | 3 | 0 | K.NLQEAEEWYK.S (Ions score 14) |
| 282–291 | 655.3088 | 1308.6031 | 1308.5986 | 3 | 0 | K.NLQEAEEWYK.S (Ions score 16) |
| 282–291 | 655.3088 | 1308.6031 | 1308.5986 | 3 | 0 | K.NLQEAEEWYK.S (Ions score 24) |

**Table S2.** *Cont.*

| 282–291 | 655.3089 | 1308.6032 | 1308.5986 | 4 | 0 | K.NLQEAEEWYK.S (Ions score 11) |
| --- | --- | --- | --- | --- | --- | --- |
| 282–291 | 655.3090 | 1308.6034 | 1308.5986 | 4 | 0 | K.NLQEAEEWYK.S (Ions score 27) |
| 282–291 | 655.3091 | 1308.6037 | 1308.5986 | 4 | 0 | K.NLQEAEEWYK.S (Ions score 22) |
| 282–291 | 655.3094 | 1308.6042 | 1308.5986 | 4 | 0 | K.NLQEAEEWYK.S (Ions score 9) |
| 282–291 | 655.3094 | 1308.6043 | 1308.5986 | 4 | 0 | K.NLQEAEEWYK.S (Ions score 11) |
| 282–291 | 655.3099 | 1308.6052 | 1308.5986 | 5 | 0 | K.NLQEAEEWYK.S (Ions score 2) |
| 282–291 | 655.3101 | 1308.6056 | 1308.5986 | 5 | 0 | K.NLQEAEEWYK.S (Ions score 9) |
| 282–291 | 655.3111 | 1308.6076 | 1308.5986 | 7 | 0 | K.NLQEAEEWYK.S (Ions score 5) |
| 292–303 | 655.8225 | 1309.6303 | 1309.6262 | 3 | 1 | K.SKYADLSDAANR.N (Ions score 66) |
| 292–303 | 437.5509 | 1309.6308 | 1309.6262 | 3 | 1 | K.SKYADLSDAANR.N (Ions score 45) |
| 292–309 | 508.5070 | 2029.9991 | 2029.9929 | 3 | 2 | K.SKYADLSDAANRNHEALR.Q (Ions score 29) |
| 294–303 | 548.2583 | 1094.502 | 1094.4992 | 3 | 0 | K.YADLSDAANR.N (Ions score 32) |
| 294–303 | 548.2585 | 1094.5025 | 1094.4992 | 3 | 0 | K.YADLSDAANR.N (Ions score 11) |
| 294–303 | 548.2587 | 1094.5028 | 1094.4992 | 3 | 0 | K.YADLSDAANR.N (Ions score 52) |
| 294–303 | 548.2590 | 1094.5034 | 1094.4992 | 4 | 0 | K.YADLSDAANR.N (Ions score 54) |
| 294–303 | 548.2592 | 1094.5038 | 1094.4992 | 4 | 0 | K.YADLSDAANR.N (Ions score 50) |
| 294–303 | 548.2609 | 1094.5072 | 1094.4992 | 7 | 0 | K.YADLSDAANR.N (Ions score 5) |
| 294–309 | 605.9645 | 1814.8718 | 1814.8659 | 3 | 1 | K.YADLSDAANRNHEALR.Q (Ions score 36) |
| 294–309 | 454.7253 | 1814.8723 | 1814.8659 | 3 | 1 | K.YADLSDAANRNHEALR.Q (Ions score 27) |
| 294–309 | 908.4435 | 1814.8725 | 1814.8659 | 4 | 1 | K.YADLSDAANRNHEALR.Q (Ions score 22) |
| 320–333 | 558.9592 | 1673.8557 | 1673.8519 | 2 | 1 | R.RQIQSLTCEVDGLR.G (Ions score 21) |
| 320–333 | 558.9596 | 1673.8569 | 1673.8519 | 3 | 1 | R.RQIQSLTCEVDGLR.G (Ions score 32) |
| 320–333 | 558.9600 | 1673.8581 | 1673.8519 | 4 | 1 | R.RQIQSLTCEVDGLR.G (Ions score 43) |
| 320–333 | 837.9364 | 1673.8582 | 1673.8519 | 4 | 1 | R.RQIQSLTCEVDGLR.G (Ions score 48) |
| 321–333 | 759.8834 | 1517.7522 | 1517.7508 | 1 | 0 | R.QIQSLTCEVDGLR.G (Ions score 28) |
| 321–333 | 759.8840 | 1517.7535 | 1517.7508 | 2 | 0 | R.QIQSLTCEVDGLR.G (Ions score 11) |
| 321–333 | 759.8851 | 1517.7557 | 1517.7508 | 3 | 0 | R.QIQSLTCEVDGLR.G (Ions score 59) |
| 334–341 | 437.2449 | 872.4752 | 872.4716 | 4 | 0 | R.GTNEALLR.Q (Ions score 56) |
| 364–369 | 394.7125 | 787.4104 | 787.4075 | 4 | 0 | R.LEEELR.Q (Ions score 30) |
| 370–377 | 335.5125 | 1003.5156 | 1003.512 | 4 | 1 | R.QLKEEMAR.H (Ions score 6) |
| 378–389 | 514.6204 | 1540.8394 | 1540.8361 | 2 | 1 | R.HLREYQELLNVK.M (Ions score 13) |

**Table S2.** *Cont.*

| 381–389 | 568.3044 | 1134.5942 | 1134.5921 | 2 | 0 | R.EYQELLNVK.M (Ions score 39) |
| --- | --- | --- | --- | --- | --- | --- |
| 381–389 | 568.3047 | 1134.5948 | 1134.5921 | 2 | 0 | R.EYQELLNVK.M (Ions score 40) |
| 381–389 | 568.3051 | 1134.5957 | 1134.5921 | 3 | 0 | R.EYQELLNVK.M (Ions score 49) |
| 381–389 | 568.3067 | 1134.5988 | 1134.5921 | 6 | 0 | R.EYQELLNVK.M (Ions score 14) |
| 381–389 | 590.7911 | 1179.5676 | 1179.5771 | −8 | 0 | R.EYQELLNVK.M Nitro (Y) (Ions score 2) |
| 390–400 | 656.3363 | 1310.658 | 1310.654 | 3 | 0 | K.MALDIEIATYR.K Oxidation (M) (Ions score 62) |
| 390–401 | 720.3832 | 1438.7518 | 1438.749 | 2 | 1 | K.MALDIEIATYRK.L Oxidation (M) (Ions score 49) |
| 390–401 | 720.3832 | 1438.7519 | 1438.749 | 2 | 1 | K.MALDIEIATYRK.L Oxidation (M) (Ions score 64) |
| 390–401 | 480.5919 | 1438.7539 | 1438.749 | 3 | 1 | K.MALDIEIATYRK.L Oxidation (M) (Ions score 37) |
| 401–409 | 530.7875 | 1059.5605 | 1059.556 | 4 | 1 | R.KLLEGEESR.I (Ions score 48) |
| 410–423 | 742.9290 | 1483.8435 | 1483.8399 | 2 | 0 | R.ISVPVHSFASLSLK.T (Ions score 58) |
| 410–423 | 495.6218 | 1483.8437 | 1483.8399 | 3 | 0 | R.ISVPVHSFASLSLK.T (Ions score 22) |
| 424–438 | 839.9078 | 1677.801 | 1677.7958 | 3 | 0 | K.TTVPEVEPPQDSHSR.K (Ions score 25) |
| 424–438 | 839.9080 | 1677.8015 | 1677.7958 | 3 | 0 | K.TTVPEVEPPQDSHSR.K (Ions score 37) |
| 424–438 | 560.2745 | 1677.8018 | 1677.7958 | 4 | 0 | K.TTVPEVEPPQDSHSR.K (Ions score 13) |
| 424–438 | 560.2747 | 1677.8023 | 1677.7958 | 4 | 0 | K.TTVPEVEPPQDSHSR.K (Ions score 36) |
| 424–438 | 560.2748 | 1677.8027 | 1677.7958 | 4 | 0 | K.TTVPEVEPPQDSHSR.K (Ions score 29) |
| 424–438 | 560.2750 | 1677.8031 | 1677.7958 | 4 | 0 | K.TTVPEVEPPQDSHSR.K (Ions score 8) |
| 424–438 | 839.9088 | 1677.8031 | 1677.7958 | 4 | 0 | K.TTVPEVEPPQDSHSR.K (Ions score 38) |
| 424–439 | 903.9560 | 1805.8974 | 1805.8908 | 4 | 1 | K.TTVPEVEPPQDSHSRK.M (Ions score 38) |
| 424–439 | 452.4818 | 1805.898 | 1805.8908 | 4 | 1 | K.TTVPEVEPPQDSHSRK.M (Ions score 17) |
| 439–444 | 380.2504 | 758.4863 | 758.4836 | 4 | 1 | R.KMVLIR.T (Ions score 36) |
| 439–444 | 388.2481 | 774.4817 | 774.4786 | 4 | 1 | R.KMVLIR.T Oxidation (M) (Ions score 24) |
| 440–444 | 316.2025 | 630.3905 | 630.3887 | 3 | 0 | K.MVLIR.T (Ions score 20) |
| 445–449 | 310.1755 | 618.3365 | 618.3337 | 5 | 0 | R.TIETR.D (Ions score 17) |
| 450–467 | 515.2477 | 2056.9619 | 2056.9549 | 3 | 1 | R.DGEVVTESQKEQHSELDK.S (Ions score 9) |
| 450–467 | 686.6614 | 2056.9625 | 2056.9549 | 4 | 1 | R.DGEVVTESQKEQHSELDK.S (Ions score 25) |
| 450–473 | 683.8232 | 2731.2636 | 2731.2573 | 2 | 2 | R.DGEVVTESQKEQHSELDKSSIHSY—(Ions score 8) |
| 450–473 | 911.4293 | 2731.266 | 2731.2573 | 3 | 2 | R.DGEVVTESQKEQHSELDKSSIHSY—(Ions score 28) |
| 460–473 | 830.3872 | 1658.7599 | 1658.7536 | 4 | 1 | K.EQHSELDKSSIHSY—(Ions score 39) |
| 460–473 | 553.9272 | 1658.7599 | 1658.7536 | 4 | 1 | K.EQHSELDKSSIHSY—(Ions score 39) |
| 460–473 | 415.6973 | 1658.7602 | 1658.7536 | 4 | 1 | K.EQHSELDKSSIHSY—(Ions score 15) |

**Table S3.** Identified tryptic peptides in sample 3.

| gi\|129822. RecName: Full=Peripherin | | | | | | |
| --- | --- | --- | --- | --- | --- | --- |
| Proteins matching the same set of peptides | | | | | | |
| gi\|149032093. peripherin 1, isoform CRA_b | | | | | | |
| **Start–End** | **Observed** | **Mr (expt)** | **Mr (calc)** | **ppm** | **Miss** | **Sequence** |
| 19–39 | 729.0294 | 2184.0663 | 2184.06 | 3 | 1 | R.RTFGPPPSLSPGAFSYSSSSR.F (Ions score 71) |
| 20–39 | 676.9952 | 2027.9639 | 2027.9589 | 2 | 0 | R.TFGPPPSLSPGAFSYSSSSR.F (Ions score 44) |
| 20–39 | 1014.9894 | 2027.9642 | 2027.9589 | 3 | 0 | R.TFGPPPSLSPGAFSYSSSSR.F (Ions score 63) |
| 20–39 | 1014.9894 | 2027.9643 | 2027.9589 | 3 | 0 | R.TFGPPPSLSPGAFSYSSSSR.F (Ions score 70) |
| 20–39 | 1014.9907 | 2027.9668 | 2027.9589 | 4 | 0 | R.TFGPPPSLSPGAFSYSSSSR.F (Ions score 74) |
| 20–39 | 1014.9912 | 2027.9679 | 2027.9589 | 4 | 0 | R.TFGPPPSLSPGAFSYSSSSR.F (Ions score 81) |
| 45–56 | 599.7784 | 1197.5423 | 1197.5391 | 3 | 0 | R.LLGSGSPSSSAR.L Phospho (ST) (Ions score 44) |
| 65–74 | 357.209 | 1068.6051 | 1068.604 | 1 | 1 | R.AGALRLPSER.L (Ions score 10) |
| 65–74 | 535.3102 | 1068.6058 | 1068.604 | 2 | 1 | R.AGALRLPSER.L (Ions score 22) |
| 65–74 | 357.2092 | 1068.6059 | 1068.604 | 2 | 1 | R.AGALRLPSER.L (Ions score 28) |
| 75–91 | 657.9897 | 1970.9472 | 1970.9407 | 3 | 0 | R.LDFSMAEALNQEFLATR.S Oxidation (M) (Ions score 24) |
| 75–91 | 986.481 | 1970.9475 | 1970.9407 | 3 | 0 | R.LDFSMAEALNQEFLATR.S Oxidation (M) (Ions score 60) |
| 75–91 | 986.4816 | 1970.9487 | 1970.9407 | 4 | 0 | R.LDFSMAEALNQEFLATR.S Oxidation (M) (Ions score 55) |
| 92–104 | 801.8918 | 1601.7691 | 1601.7645 | 3 | 1 | R.SNEKQELQELNDR.F (Ions score 71) |
| 92–104 | 534.9304 | 1601.7694 | 1601.7645 | 3 | 1 | R.SNEKQELQELNDR.F (Ions score 43) |
| 105–111 | 434.7329 | 867.4512 | 867.449 | 3 | 0 | R.FANFIEK.V (Ions score 16) |
| 105–111 | 434.7331 | 867.4516 | 867.449 | 3 | 0 | R.FANFIEK.V (Ions score 39) |
| 114–123 | 595.3215 | 1188.6284 | 1188.6251 | 3 | 0 | R.FLEQQNAALR.G (Ions score 62) |
| 114–130 | 644.3433 | 1930.008 | 1930.002 | 3 | 1 | R.FLEQQNAALRGELSQAR.G (Ions score 43) |
| 114–130 | 966.0117 | 1930.0088 | 1930.002 | 3 | 1 | R.FLEQQNAALRGELSQAR.G (Ions score 15) |
| 131–146 | 633.6444 | 1897.9114 | 1897.9064 | 3 | 1 | R.GQEPARADQLCQQELR.E (Ions score 22) |
| 131–146 | 949.9631 | 1897.9117 | 1897.9064 | 3 | 1 | R.GQEPARADQLCQQELR.E (Ions score 34) |
| 137–146 | 630.8054 | 1259.5962 | 1259.5928 | 3 | 0 | R.ADQLCQQELR.E (Ions score 48) |
| 150–157 | 493.2942 | 984.5739 | 984.5716 | 2 | 1 | R.RELELLGR.E (Ions score 42) |
| 151–157 | 415.2438 | 828.473 | 828.4705 | 3 | 0 | R.ELELLGR.E (Ions score 25) |
| 151–157 | 415.2439 | 828.4732 | 828.4705 | 3 | 0 | R.ELELLGR.E (Ions score 31) |
| 160–166 | 451.2478 | 900.4811 | 900.4777 | 4 | 1 | R.DRVQVER.D (Ions score 3) |

**Table S3.** *Cont.*

| 160–177 | 496.7683 | 1983.044 | 1983.0385 | 3 | 2 | R.DRVQVERDGLAEDLGALK.Q (Ions score 14) |
| --- | --- | --- | --- | --- | --- | --- |
| 160–177 | 662.0226 | 1983.0459 | 1983.0385 | 4 | 2 | R.DRVQVERDGLAEDLGALK.Q (Ions score 23) |
| 162–177 | 571.6454 | 1711.9145 | 1711.9104 | 2 | 1 | R.VQVERDGLAEDLGALK.Q (Ions score 35) |
| 162–177 | 571.6459 | 1711.916 | 1711.9104 | 3 | 1 | R.VQVERDGLAEDLGALK.Q (Ions score 31) |
| 167–177 | 551.2949 | 1100.5753 | 1100.5713 | 4 | 0 | R.DGLAEDLGALK.Q (Ions score 55) |
| 167–179 | 462.5854 | 1384.7345 | 1384.731 | 3 | 1 | R.DGLAEDLGALKQR.L (Ions score 3) |
| 167–179 | 462.5858 | 1384.7354 | 1384.731 | 3 | 1 | R.DGLAEDLGALKQR.L (Ions score 13) |
| 167–179 | 693.3752 | 1384.7359 | 1384.731 | 4 | 1 | R.DGLAEDLGALKQR.L (Ions score 59) |
| 186–198 | 407.474 | 1625.8669 | 1625.8638 | 2 | 2 | R.KREDAEHNLVLFR.K (Ions score 27) |
| 186–198 | 542.9629 | 1625.867 | 1625.8638 | 2 | 2 | R.KREDAEHNLVLFR.K (Ions score 25) |
| 186–198 | 542.9632 | 1625.8678 | 1625.8638 | 2 | 2 | R.KREDAEHNLVLFR.K (Ions score 11) |
| 186–198 | 813.9412 | 1625.8679 | 1625.8638 | 3 | 2 | R.KREDAEHNLVLFR.K (Ions score 52) |
| 186–198 | 407.4745 | 1625.869 | 1625.8638 | 3 | 2 | R.KREDAEHNLVLFR.K (Ions score 25) |
| 186–198 | 407.4747 | 1625.8696 | 1625.8638 | 4 | 2 | R.KREDAEHNLVLFR.K (Ions score 11) |
| 187–198 | 500.2647 | 1497.7722 | 1497.7688 | 2 | 1 | K.REDAEHNLVLFR.K (Ions score 15) |
| 188–198 | 448.2301 | 1341.6686 | 1341.6677 | 1 | 0 | R.EDAEHNLVLFR.K (Ions score 17) |
| 188–198 | 671.8441 | 1341.6736 | 1341.6677 | 4 | 0 | R.EDAEHNLVLFR.K (Ions score 67) |
| 188–199 | 490.9295 | 1469.7668 | 1469.7626 | 3 | 1 | R.EDAEHNLVLFRK.D (Ions score 28) |
| 200–213 | 544.6143 | 1630.8211 | 1630.8162 | 3 | 1 | K.DVDDATLSRLELER.K (Ions score 48) |
| 200–213 | 816.418 | 1630.8214 | 1630.8162 | 3 | 1 | K.DVDDATLSRLELER.K (Ions score 28) |
| 214–226 | 537.618 | 1609.8321 | 1609.8273 | 3 | 1 | R.KIESLMDEIEFLK.K Oxidation (M) (Ions score 32) |
| 214–226 | 805.9236 | 1609.8327 | 1609.8273 | 3 | 1 | R.KIESLMDEIEFLK.K Oxidation (M) (Ions score 71) |
| 214–227 | 574.9847 | 1721.9324 | 1721.9273 | 3 | 2 | R.KIESLMDEIEFLKK.L (Ions score 48) |
| 214–227 | 435.4891 | 1737.9272 | 1737.9222 | 3 | 2 | R.KIESLMDEIEFLKK.L Oxidation (M) (Ions score 13) |
| 214–227 | 869.971 | 1737.9275 | 1737.9222 | 3 | 2 | R.KIESLMDEIEFLKK.L Oxidation (M) (Ions score 65) |
| 214–227 | 580.3165 | 1737.9278 | 1737.9222 | 3 | 2 | R.KIESLMDEIEFLKK.L Oxidation (M) (Ions score 39) |
| 215–226 | 741.8758 | 1481.737 | 1481.7323 | 3 | 0 | K.IESLMDEIEFLK.K Oxidation (M) (Ions score 53) |
| 215–227 | 537.6185 | 1609.8336 | 1609.8273 | 4 | 1 | K.IESLMDEIEFLKK.L Oxidation (M) (Ions score 12) |
| 276–285 | 655.3071 | 1308.5996 | 1308.5986 | 1 | 0 | K.NLQEAEEWYK.S (Ions score 27) |
| 276–285 | 655.309 | 1308.6035 | 1308.5986 | 4 | 0 | K.NLQEAEEWYK.S (Ions score 39) |

**Table S3.** *Cont.*

| 286–297 | 437.5507 | 1309.6304 | 1309.6262 | 3 | 1 | K.SKYADLSDAANR.N (Ions score 39) |
| --- | --- | --- | --- | --- | --- | --- |
| 286–297 | 655.8226 | 1309.6307 | 1309.6262 | 3 | 1 | K.SKYADLSDAANR.N (Ions score 36) |
| 288–297 | 548.2579 | 1094.5012 | 1094.4992 | 2 | 0 | K.YADLSDAANR.N (Ions score 55) |
| 288–303 | 454.7248 | 1814.8702 | 1814.8659 | 2 | 1 | K.YADLSDAANRNHEALR.Q (Ions score 15) |
| 288–303 | 605.9642 | 1814.8707 | 1814.8659 | 3 | 1 | K.YADLSDAANRNHEALR.Q (Ions score 39) |
| 288–303 | 908.443 | 1814.8714 | 1814.8659 | 3 | 1 | K.YADLSDAANRNHEALR.Q (Ions score 32) |
| 314–327 | 837.9353 | 1673.856 | 1673.8519 | 2 | 1 | R.RQIQSLTCEVDGLR.G (Ions score 48) |
| 314–327 | 558.9593 | 1673.8562 | 1673.8519 | 3 | 1 | R.RQIQSLTCEVDGLR.G (Ions score 35) |
| 315–327 | 759.8843 | 1517.7541 | 1517.7508 | 2 | 0 | R.QIQSLTCEVDGLR.G (Ions score 3) |
| 315–327 | 759.8845 | 1517.7545 | 1517.7508 | 2 | 0 | R.QIQSLTCEVDGLR.G (Ions score 64) |
| 364–371 | 502.7618 | 1003.5089 | 1003.512 | −3 | 1 | R.QLKEEMAR.H (Ions score 10) |
| 375–383 | 568.3044 | 1134.5942 | 1134.5921 | 2 | 0 | R.EYQELLNVK.M (Ions score 32) |
| 384–394 | 656.3362 | 1310.6578 | 1310.654 | 3 | 0 | K.MALDIEIATYR.K Oxidation (M) (Ions score 47) |
| 384–395 | 480.5915 | 1438.7526 | 1438.749 | 2 | 1 | K.MALDIEIATYRK.L Oxidation (M) (Ions score 42) |
| 404–417 | 495.6222 | 1483.8447 | 1483.8399 | 3 | 0 | R.ISVPVHSFASLSLK.T (Ions score 25) |
| 418–432 | 839.9073 | 1677.8001 | 1677.7958 | 3 | 0 | K.TTVPEVEPPQDSHSR.K (Ions score 29) |
| 418–432 | 560.2741 | 1677.8005 | 1677.7958 | 3 | 0 | K.TTVPEVEPPQDSHSR.K (Ions score 14) |
| 418–433 | 452.4812 | 1805.8957 | 1805.8908 | 3 | 1 | K.TTVPEVEPPQDSHSRK.M (Ions score 16) |
| 418–433 | 602.9725 | 1805.8958 | 1805.8908 | 3 | 1 | K.TTVPEVEPPQDSHSRK.M (Ions score 13) |
| 418–433 | 903.9557 | 1805.8968 | 1805.8908 | 3 | 1 | K.TTVPEVEPPQDSHSRK.M (Ions score 37) |
| 433–438 | 388.2476 | 774.4806 | 774.4786 | 3 | 1 | R.KMVLIR.T Oxidation (M) (Ions score 19) |
| 434–438 | 316.2023 | 630.39 | 630.3887 | 2 | 0 | K.MVLIR.T (Ions score 18) |
| 439–454 | 633.9678 | 1898.8817 | 1898.8986 | −9 | 2 | R.TIETRDGEKVVTESQK.E Phospho (ST) (Ions score 15) |
| 448–462 | 586.2976 | 1755.8708 | 1755.8639 | 4 | 1 | K.VVTESQKEQHSELDK.S (Ions score 7) |
| 448–468 | 608.5502 | 2430.1718 | 2430.1663 | 2 | 2 | K.VVTESQKEQHSELDKSSIHSY—(Ions score 14) |
| 448–468 | 811.0648 | 2430.1725 | 2430.1663 | 3 | 2 | K.VVTESQKEQHSELDKSSIHSY—(Ions score 27) |
| 448–468 | 1216.0935 | 2430.1725 | 2430.1663 | 3 | 2 | K.VVTESQKEQHSELDKSSIHSY—(Ions score 51) |
| 455–468 | 830.3861 | 1658.7577 | 1658.7536 | 2 | 1 | K.EQHSELDKSSIHSY—(Ions score 43) |
| 455–468 | 415.6968 | 1658.7583 | 1658.7536 | 3 | 1 | K.EQHSELDKSSIHSY—(Ions score 9) |
| 455–468 | 553.9267 | 1658.7583 | 1658.7536 | 3 | 1 | K.EQHSELDKSSIHSY—(Ions score 31) |

**Table S4.** Identified tryptic peptides in sample 4.

| gi\|129822 RecName: Full=Peripherin | | | | | | |
| --- | --- | --- | --- | --- | --- | --- |
| Proteins matching the same set of peptides | | | | | | |
| gi\|149032093 peripherin 1, isoform CRA_b [*Rattus norvegicus*] | | | | | | |
| **Start–End** | **Observed** | **Mr (expt)** | **Mr (calc)** | **ppm** | **Miss** | **Sequence** |
| 19–39 | 729.0292 | 2184.0659 | 2184.06 | 3 | 1 | R.RTFGPPPSLSPGAFSYSSSSR.F (Ions score 40) |
| 20–39 | 1014.9886 | 2027.9626 | 2027.9589 | 2 | 0 | R.TFGPPPSLSPGAFSYSSSSR.F (Ions score 62) |
| 20–39 | 1014.9889 | 2027.9632 | 2027.9589 | 2 | 0 | R.TFGPPPSLSPGAFSYSSSSR.F (Ions score 45) |
| 20–39 | 1014.9889 | 2027.9632 | 2027.9589 | 2 | 0 | R.TFGPPPSLSPGAFSYSSSSR.F (Ions score 38) |
| 20–39 | 676.9952 | 2027.9637 | 2027.9589 | 2 | 0 | R.TFGPPPSLSPGAFSYSSSSR.F (Ions score 42) |
| 20–39 | 1014.9922 | 2027.9698 | 2027.9589 | 5 | 0 | R.TFGPPPSLSPGAFSYSSSSR.F (Ions score 41) |
| 45–56 | 599.7784 | 1197.5423 | 1197.5391 | 3 | 0 | R.LLGSGSPSSSAR.L Phospho (ST) (Ions score 47) |
| 65–74 | 357.2097 | 1068.6073 | 1068.604 | 3 | 1 | R.AGALRLPSER.L (Ions score 26) |
| 75–91 | 986.4803 | 1970.946 | 1970.9407 | 3 | 0 | R.LDFSMAEALNQEFLATR.S Oxidation (M) (Ions score 46) |
| 75–91 | 986.4806 | 1970.9466 | 1970.9407 | 3 | 0 | R.LDFSMAEALNQEFLATR.S Oxidation (M) (Ions score 51) |
| 92–104 | 801.8917 | 1601.7688 | 1601.7645 | 3 | 1 | R.SNEKQELQELNDR.F (Ions score 71) |
| 92–104 | 534.9304 | 1601.7693 | 1601.7645 | 3 | 1 | R.SNEKQELQELNDR.F (Ions score 48) |
| 105–111 | 434.733 | 867.4514 | 867.449 | 3 | 0 | R.FANFIEK.V (Ions score 39) |
| 114–123 | 595.3217 | 1188.6288 | 1188.6251 | 3 | 0 | R.FLEQQNAALR.G (Ions score 63) |
| 131–146 | 633.6446 | 1897.9119 | 1897.9064 | 3 | 1 | R.GQEPARADQLCQQELR.E (Ions score 26) |
| 137–146 | 630.8055 | 1259.5964 | 1259.5928 | 3 | 0 | R.ADQLCQQELR.E (Ions score 41) |
| 150–157 | 493.2943 | 984.5741 | 984.5716 | 3 | 1 | R.RELELLGR.E (Ions score 38) |
| 151–157 | 415.2433 | 828.4721 | 828.4705 | 2 | 0 | R.ELELLGR.E (Ions score 28) |
| 160–166 | 451.2478 | 900.481 | 900.4777 | 4 | 1 | R.DRVQVER.D (Ions score 4) |
| 162–166 | 315.6832 | 629.3519 | 629.3497 | 4 | 0 | R.VQVER.D (Ions score 7) |
| 167–177 | 551.2949 | 1100.5753 | 1100.5713 | 4 | 0 | R.DGLAEDLGALK.Q (Ions score 62) |
| 167–179 | 693.3736 | 1384.7326 | 1384.731 | 1 | 1 | R.DGLAEDLGALKQR.L (Ions score 44) |
| 167–179 | 462.5852 | 1384.7338 | 1384.731 | 2 | 1 | R.DGLAEDLGALKQR.L (Ions score 18) |

**Table S4.** *Cont.*

| 180–185 | 388.6942 | | 775.3739 | 775.3712 | 4 | 0 | R.LEEETR.K (Ions score 11) |
| --- | --- | --- | --- | --- | --- | --- | --- |
| 186–198 | 407.474 | 1625.867 | | 1625.8638 | 2 | 2 | R.KREDAEHNLVLFR.K (Ions score 3) |
| 186–198 | 542.9633 | 1625.8681 | | 1625.8638 | 3 | 2 | R.KREDAEHNLVLFR.K (Ions score 21) |
| 186–198 | 407.4743 | 1625.8682 | | 1625.8638 | 3 | 2 | R.KREDAEHNLVLFR.K (Ions score 32) |
| 186–198 | 407.4745 | 1625.8689 | | 1625.8638 | 3 | 2 | R.KREDAEHNLVLFR.K (Ions score 18) |
| 186–198 | 813.9417 | 1625.8689 | | 1625.8638 | 3 | 2 | R.KREDAEHNLVLFR.K (Ions score 55) |
| 187–198 | 500.265 | 1497.7731 | | 1497.7688 | 3 | 1 | K.REDAEHNLVLFR.K (Ions score 22) |
| 188–198 | 448.2307 | 1341.6702 | | 1341.6677 | 2 | 0 | R.EDAEHNLVLFR.K (Ions score 22) |
| 188–198 | 671.8429 | 1341.6712 | | 1341.6677 | 3 | 0 | R.EDAEHNLVLFR.K (Ions score 51) |
| 200–213 | 816.4163 | 1630.8181 | | 1630.8162 | 1 | 1 | K.DVDDATLSRLELER.K (Ions score 25) |
| 200–213 | 544.6138 | 1630.8195 | | 1630.8162 | 2 | 1 | K.DVDDATLSRLELER.K (Ions score 33) |
| 200–213 | 816.4173 | 1630.82 | | 1630.8162 | 2 | 1 | K.DVDDATLSRLELER.K (Ions score 24) |
| 200–213 | 816.4179 | 1630.8211 | | 1630.8162 | 3 | 1 | K.DVDDATLSRLELER.K (Ions score 23) |
| 214–226 | 805.9224 | 1609.8303 | | 1609.8273 | 2 | 1 | R.KIESLMDEIEFLK.K Oxidation (M) (Ions score 69) |
| 214–226 | 537.6176 | 1609.8308 | | 1609.8273 | 2 | 1 | R.KIESLMDEIEFLK.K Oxidation (M) (Ions score 25) |
| 214–226 | 805.9234 | 1609.8322 | | 1609.8273 | 3 | 1 | R.KIESLMDEIEFLK.K Oxidation (M) (Ions score 85) |
| 214–227 | 574.9849 | 1721.9329 | | 1721.9273 | 3 | 2 | R.KIESLMDEIEFLKK.L (Ions score 27) |
| 214–227 | 435.4886 | 1737.9253 | | 1737.9222 | 2 | 2 | R.KIESLMDEIEFLKK.L Oxidation (M) (Ions score 2) |
| 214–227 | 580.3165 | 1737.9278 | | 1737.9222 | 3 | 2 | R.KIESLMDEIEFLKK.L Oxidation (M) (Ions score 49) |
| 215–226 | 741.8768 | 1481.739 | | 1481.7323 | 5 | 0 | K.IESLMDEIEFLK.K Oxidation (M) (Ions score 36) |
| 215–227 | 537.6177 | 1609.8314 | | 1609.8273 | 3 | 1 | K.IESLMDEIEFLKK.L Oxidation (M) (Ions score 10) |
| 276–285 | 655.3083 | 1308.602 | | 1308.5986 | 3 | 0 | K.NLQEAEEWYK.S (Ions score 46) |
| 288–297 | 548.2589 | 1094.5033 | | 1094.4992 | 4 | 0 | K.YADLSDAANR.N (Ions score 64) |
| 288–303 | 454.7252 | 1814.8717 | | 1814.8659 | 3 | 1 | K.YADLSDAANRNHEALR.Q (Ions score 7) |
| 288–303 | 605.9647 | 1814.8722 | | 1814.8659 | 3 | 1 | K.YADLSDAANRNHEALR.Q (Ions score 27) |
| 288–303 | 908.4437 | 1814.8728 | | 1814.8659 | 4 | 1 | K.YADLSDAANRNHEALR.Q (Ions score 27) |
| 314–327 | 837.9354 | 1673.8563 | | 1673.8519 | 3 | 1 | R.RQIQSLTCEVDGLR.G (Ions score 38) |
| 314–327 | 558.9603 | 1673.859 | | 1673.8519 | 4 | 1 | R.RQIQSLTCEVDGLR.G (Ions score 39) |
| 375–383 | 568.3065 | 1134.5985 | | 1134.5921 | 6 | 0 | R.EYQELLNVK.M (Ions score 48) |

**Table S4.** *Cont.*

| 384–394 | 656.3364 | 1310.6582 | 1310.654 | 3 | 0 | K.MALDIEIATYR.K Oxidation (M) (Ions score 60) |
| --- | --- | --- | --- | --- | --- | --- |
| 384–395 | 480.591 | 1438.7512 | 1438.749 | 2 | 1 | K.MALDIEIATYRK.L Oxidation (M) (Ions score 24) |
| 404–417 | 495.6219 | 1483.8439 | 1483.8399 | 3 | 0 | R.ISVPVHSFASLSLK.T (Ions score 13) |
| 418–432 | 560.2742 | 1677.8009 | 1677.7958 | 3 | 0 | K.TTVPEVEPPQDSHSR.K (Ions score 17) |
| 418–432 | 839.908 | 1677.8015 | 1677.7958 | 3 | 0 | K.TTVPEVEPPQDSHSR.K (Ions score 20) |
| 434–438 | 316.2024 | 630.3903 | 630.3887 | 3 | 0 | K.MVLIR.T (Ions score 12) |
| 434–438 | 316.2025 | 630.3905 | 630.3887 | 3 | 0 | K.MVLIR.T (Ions score 26) |
| 439–454 | 633.9682 | 1898.8828 | 1898.8986 | −8 | 2 | R.TIETRDGEKVVTESQK.E Phospho (ST) (Ions score 8) |
| 448–462 | 586.2976 | 1755.871 | 1755.8639 | 4 | 1 | K.VVTESQKEQHSELDK.S (Ions score 0) |
| 448–468 | 608.5505 | 2430.1728 | 2430.1663 | 3 | 2 | K.VVTESQKEQHSELDKSSIHSY—(Ions score 16) |
| 448–468 | 811.0653 | 2430.1741 | 2430.1663 | 3 | 2 | K.VVTESQKEQHSELDKSSIHSY—(Ions score 35) |
| 455–468 | 553.9265 | 1658.7577 | 1658.7536 | 2 | 1 | K.EQHSELDKSSIHSY—(Ions score 24) |

**Table S5.** Identified tryptic peptides in sample 5.

| gi\|129822 RecName: Full=Peripherin | | | | | | |
| --- | --- | --- | --- | --- | --- | --- |
| Proteins matching the same set of peptides | | | | | | |
| gi\|149032093 peripherin 1, isoform CRA_b [*Rattus norvegicus*] | | | | | | |
| **Start–End** | **Observed** | **Mr (expt)** | **Mr (calc)** | **ppm** | **Miss** | **Sequence** |
| 20–39 | 676.9953 | 2027.9641 | 2027.9589 | 3 | 0 | R.TFGPPPSLSPGAFSYSSSSR.F (Ions score 23) |
| 20–39 | 676.9963 | 2027.9672 | 2027.9589 | 4 | 0 | R.TFGPPPSLSPGAFSYSSSSR.F (Ions score 21) |
| 65–74 | 357.2097 | 1068.6072 | 1068.604 | 3 | 1 | R.AGALRLPSER.L (Ions score 24) |
| 65–74 | 535.3113 | 1068.6081 | 1068.604 | 4 | 1 | R.AGALRLPSER.L (Ions score 20) |
| 75–91 | 986.4808 | 1970.9471 | 1970.9407 | 3 | 0 | R.LDFSMAEALNQEFLATR.S Oxidation (M) (Ions score 42) |
| 92–104 | 534.9302 | 1601.7689 | 1601.7645 | 3 | 1 | R.SNEKQELQELNDR.F (Ions score 43) |
| 92–104 | 801.892 | 1601.7695 | 1601.7645 | 3 | 1 | R.SNEKQELQELNDR.F (Ions score 63) |
| 105–111 | 434.7328 | 867.451 | 867.449 | 2 | 0 | R.FANFIEK.V (Ions score 35) |
| 114–123 | 595.322 | 1188.6295 | 1188.6251 | 4 | 0 | R.FLEQQNAALR.G (Ions score 57) |
| 131–146 | 633.6447 | 1897.9123 | 1897.9064 | 3 | 1 | R.GQEPARADQLCQQELR.E (Ions score 21) |
| 137–146 | 630.8058 | 1259.5971 | 1259.5928 | 3 | 0 | R.ADQLCQQELR.E (Ions score 45) |

**Table S5.** *Cont.*

| 151–157 | 415.2432 | 828.4718 | 828.4705 | 2 | 0 | R.ELELLGR.E (Ions score 38) |
| --- | --- | --- | --- | --- | --- | --- |
| 151–157 | 415.2438 | 828.4731 | 828.4705 | 3 | 0 | R.ELELLGR.E (Ions score 32) |
| 167–177 | 551.2951 | 1100.5756 | 1100.5713 | 4 | 0 | R.DGLAEDLGALK.Q (Ions score 43) |
| 167–177 | 551.2954 | 1100.5763 | 1100.5713 | 4 | 0 | R.DGLAEDLGALK.Q (Ions score 60) |
| 186–198 | 542.9636 | 1625.869 | 1625.8638 | 3 | 2 | R.KREDAEHNLVLFR.K (Ions score 35) |
| 186–198 | 407.4748 | 1625.8701 | 1625.8638 | 4 | 2 | R.KREDAEHNLVLFR.K (Ions score 27) |
| 186–198 | 813.943 | 1625.8715 | 1625.8638 | 5 | 2 | R.KREDAEHNLVLFR.K (Ions score 49) |
| 188–198 | 448.2317 | 1341.6733 | 1341.6677 | 4 | 0 | R.EDAEHNLVLFR.K (Ions score 12) |
| 188–198 | 671.8445 | 1341.6745 | 1341.6677 | 5 | 0 | R.EDAEHNLVLFR.K (Ions score 30) |
| 200–213 | 544.6138 | 1630.8197 | 1630.8162 | 2 | 1 | K.DVDDATLSRLELER.K (Ions score 32) |
| 200–213 | 816.4181 | 1630.8217 | 1630.8162 | 3 | 1 | K.DVDDATLSRLELER.K (Ions score 27) |
| 214–226 | 805.9236 | 1609.8327 | 1609.8273 | 3 | 1 | R.KIESLMDEIEFLK.K Oxidation (M) (Ions score 70) |
| 214–226 | 537.6185 | 1609.8338 | 1609.8273 | 4 | 1 | R.KIESLMDEIEFLK.K Oxidation (M) (Ions score 13) |
| 214–227 | 435.4891 | 1737.9273 | 1737.9222 | 3 | 2 | R.KIESLMDEIEFLKK.L Oxidation (M) (Ions score 4) |
| 214–227 | 580.3171 | 1737.9296 | 1737.9222 | 4 | 2 | R.KIESLMDEIEFLKK.L Oxidation (M) (Ions score 49) |
| 215–226 | 733.8751 | 1465.7357 | 1465.7374 | −1 | 0 | K.IESLMDEIEFLK.K (Ions score 3) |
| 276–285 | 655.3088 | 1308.603 | 1308.5986 | 3 | 0 | K.NLQEAEEWYK.S (Ions score 41) |
| 288–297 | 548.2587 | 1094.5028 | 1094.4992 | 3 | 0 | K.YADLSDAANR.N (Ions score 47) |
| 288–303 | 908.4423 | 1814.87 | 1814.8659 | 2 | 1 | K.YADLSDAANRNHEALR.Q (Ions score 31) |
| 288–303 | 605.9641 | 1814.8703 | 1814.8659 | 2 | 1 | K.YADLSDAANRNHEALR.Q (Ions score 29) |
| 372–394 | 729.3679 | 2913.4423 | 2913.4459 | −1 | 2 | R.HLREYQELLNVKMALDIEIATYR.K Oxidation (M); Phospho (Y) (Ions score 0) |
| 375–383 | 568.3054 | 1134.5962 | 1134.5921 | 4 | 0 | R.EYQELLNVK.M (Ions score 49) |
| 404–417 | 495.6221 | 1483.8446 | 1483.8399 | 3 | 0 | R.ISVPVHSFASLSLK.T (Ions score 14) |
| 418–432 | 560.2741 | 1677.8005 | 1677.7958 | 3 | 0 | K.TTVPEVEPPQDSHSR.K (Ions score 8) |
| 418–432 | 839.9078 | 1677.8011 | 1677.7958 | 3 | 0 | K.TTVPEVEPPQDSHSR.K (Ions score 23) |
| 433–438 | 380.2504 | 758.4862 | 758.4836 | 3 | 1 | R.KMVLIR.T (Ions score 30) |
| 434–438 | 316.2024 | 630.3903 | 630.3887 | 3 | 0 | K.MVLIR.T (Ions score 18) |
| 448–468 | 608.5505 | 2430.1731 | 2430.1663 | 3 | 2 | K.VVTESQKEQHSELDKSSIHSY—(Ions score 5) |
| 448–468 | 811.0652 | 2430.1737 | 2430.1663 | 3 | 2 | K.VVTESQKEQHSELDKSSIHSY—(Ions score 28) |
